# Evolution of UV reflection in bee‐ and bird‐pollinated flowers

**DOI:** 10.1111/plb.70138

**Published:** 2025-11-14

**Authors:** L. C. Oliveira, V. L. G. Brito, K. Lunau, S. Gerten, P. E. M. Oliveira, L. R. F. Melo, F. J. Telles, P. J. Bergamo

**Affiliations:** ^1^ Programa de Pós‐Graduação em Biologia Vegetal, Instituto de Biologia Universidade Estadual de Campinas São Paulo Brazil; ^2^ Departamento de Biodiversidade, Instituto de Biociências de Rio Claro Universidade Estadual Paulista—Rio Claro São Paulo Brazil; ^3^ Instituto de Biologia Universidade Federal de Uberlândia (UFU) Uberlândia Minas Gerais Brazil; ^4^ Department Biology, Institute of Sensory Ecology Heinrich‐Heine‐University Düsseldorf Düsseldorf Germany; ^5^ Department of Ecology and Genetics Uppsala University, EBC Norbyvägen Uppsala Sweden

**Keywords:** Brownian motion, colour evolution, evolutionary models, Ornstein–Uhlenbeck, plant–pollinator interactions, sensory exclusion

## Abstract

- Floral colour is a key trait mediating plant–pollinator interactions, with UV reflection hypothesized to evolve in response to the effectiveness of pollinators. The bee‐avoidance hypothesis predicts higher UV reflection in white flowers and lower reflection in red and yellow flowers of bird‐pollinated plants, with opposite patterns in bee‐pollinated flowers. However, the macroevolutionary role of this process remains unclear.
- We analysed 245 angiosperm species using ancestral state reconstruction and comparative evolutionary models to assess UV reflection patterns in relation to flower colour and pollinator type.
- Ancestral reconstructions revealed frequent transitions in both hue and UV reflection states. Higher UV reflection optima were found in red and bee‐pollinated flowers compared to red bird‐pollinated flowers, supporting the role of UV in bee attraction. White and yellow bee‐pollinated lineages showed elevated evolutionary rates and selective strength compared to bird‐pollinated lineages of the same hue. Unexpectedly, yellow bird‐pollinated flowers displayed higher UV reflection optima than yellow bee‐pollinated flowers.
- Our results suggest that both attraction and exclusion mechanisms contribute to the macroevolution of UV reflection in flowers. We found strong support for the bee‐avoidance hypothesis in red flowers. However, while white flowers generally followed predicted patterns, yellow flowers showed a reverse pattern, with higher UV reflection in bird‐pollinated lineages, providing contrasting evidence across colour categories. These findings highlight complex and colour‐dependent selection dynamics in pollinator‐mediated floral evolution.

## Graphical abstract

Pollinators shape floral UV reflection across angiosperms, supporting the bee‐avoidance hypothesis in red flowers and revealing mixed patterns in white and yellow flowers.

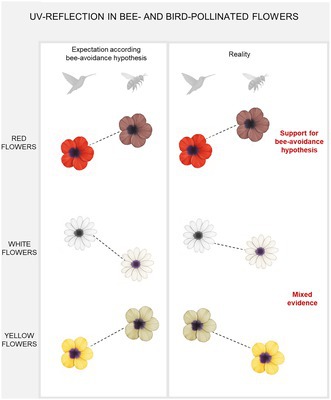

## INTRODUCTION

Flower colour plays a critical role in plant reproduction because it is one of the main signals that floral visitors use to locate flowers (Chittka & Raine 2006; Willmer 2011). Its expression depends not only on pigment composition and environmental context but also on how pollinators perceive colour, which is determined by the structure and sensitivity of their visual systems (Chittka & Thomson 2001; Arista *et al*. 2013; Van Der Kooi *et al*. 2019; Dalrymple *et al*. 2020; Garcia *et al*. 2020; Trunschke *et al*. 2021). The visual systems of floral visitors, including the specific type and number of photoreceptors, vary among and within taxonomic groups, such as insects and birds (Peitsch *et al*. 1992; Briscoe & Chittka 2001; Weiss 2001; Osorio & Vorobyev 2008; Van Der Kooi *et al*. 2019). For instance, most bee species have three photoreceptor types sensitive to UV, blue, and green light (Briscoe & Chittka 2001). In contrast, birds generally have four photoreceptor types, and perceive UV, blue, green, and red light (Ödeen & Håstad 2010; Kelber 2019). These differences in sensory perception shape how floral colours are discriminated and selected by pollinators and may drive divergent evolutionary trajectories of floral traits (Van Der Kooi *et al*. 2019). While spectral features of colour such as hue, saturation, brightness, and colour contrast have been studied in relation to pollinator preferences (Giurfa *et al*. 1995; Lunau *et al*. 1996; Dyer *et al*. 2016), the macroevolutionary consequences of these visual interactions – particularly with respect to UV reflection –remain poorly understood.

Floral colour perception is mediated by multiple spectral features: (i) hue, the dominant wavelength perceived (e.g., red, yellow; Giurfa *et al*. 1995; Dyer *et al*. 2016); (ii) saturation, the spectral purity or intensity of a colour (Lunau *et al*. 1996, but see van der Kooi & Spaethe 2022; Lunau *et al*. 2024); (iii) brightness, the total amount of light reflected (Kelber *et al*. 2002, 2003; Kelber 2005); and (iv) contrast, which determines how easily a flower stands out from its background (Dyer *et al*. 2008). Contrast can be further divided into two components. Chromatic contrast refers to the difference in colour signals processed by different photoreceptor types (i.e., hue differences), and is particularly relevant when colours are well‐separated in colour space. Achromatic contrast, on the other hand, refers to differences in brightness or intensity, often processed by a single photoreceptor type (typically the green receptor in bees). Both types of contrast affect detectability and flower discrimination at varying distances (Giurfa *et al*. 1995; Spaethe *et al*. 2001; Van Der Kooi *et al*. 2019). In this context, flowers with higher saturation and stronger colour contrast against the background are often more attractive to bees and birds (Lunau *et al*. 1996; Van Der Kooi *et al*. 2019; Stoddard *et al*. 2020; Lunau & Dyer 2024), enhancing flower visitation and plant reproduction (Chen *et al*. 2020). Furthermore, ultraviolet (UV) reflection in flowers is of special interest because it strongly interacts with bee visual systems (high UV sensitivity) and varies in salience to bird visual systems, potentially affecting pollinator attraction (Chittka *et al*. 1994; Kevan *et al*. 2001; Stoddard *et al*. 2020). Additionally, UV‐absorbing pigments may play a protective role against abiotic stressors, such as UV radiation, by shielding reproductive structures from damage (Koski & Ashman 2016). This dual role suggests that both biotic and abiotic selective pressures can influence the evolution of floral UV reflectance (Koski & Ashman 2015a, 2016; Koski *et al*. 2020). The amounts of UV reflection within flowers vary between plant pollination systems. Although birds are sensitive to UV light, there is no strong association between UV reflection and bird attraction (Stoddard *et al*. 2020). In this context, the spectral sensitivity of birds to red wavelengths has been used as evidence to explain the association between birds and UV‐absorbing red flowers (Shrestha *et al*. 2013), although they do not innately prefer red colours (Stiles 1976; Lunau & Maier 1995; Lunau *et al*. 2011; Stoddard *et al*. 2020).

An alternative hypothesis suggests that the evolution of specific colours in flowers primarily pollinated by birds is a result of sensory exclusion pressures from red‐insensitive floral antagonists, which are typically bees that act as nectar robbers, pollen consumers, and/or less efficient pollinators (“bee‐avoidance hypothesis”, Raven 1972; Lunau & Maier 1995; Cronk & Ojeda 2008; Lunau *et al*. 2011). While pollinators are often emphasized as the main selective agents shaping floral traits, antagonists also exert strong and contrasting selective pressures on flower colour, morphology, and reward accessibility (Strauss & Irwin 2004). These interactions may favour floral traits that deter damage or reduce attraction to inefficient or harmful visitors. Therefore, to fully understand the evolution of floral traits, it is essential to consider the influence of both mutualistic and antagonistic interactions. The bee‐avoidance hypothesis postulates that sensorial exclusion may reduce the interference of antagonistic bees because some flowers pollinated by birds are less conspicuous to them (Rodríguez‐Gironés & Santamaría 2004; Bergamo *et al*. 2016). Therefore, such sensorial exclusion opens a private niche for effective bird pollinators to exploit, supporting the bee‐avoidance hypothesis (Lunau *et al*. 2011). This is the case of pure red flowers (i.e., that do not reflect other wavelengths) which present lower spectral purity and colour contrasts in the bee vision. On the other hand, red bee‐pollinated flowers often reflect UV, which enhances colour contrast for bees (Chen, Liu, *et al*. 2020). The bee‐avoidance hypothesis has been investigated in field and experimental studies (Lunau *et al*. 2011; Bergamo *et al*. 2016; Camargo *et al*. 2019; Chen *et al*. 2020; Rodríguez‐Sambruno *et al*. 2024). Nevertheless, it is still unclear if such pressures from less effective visitors have influenced macroevolutionary patterns of UV reflection in flowers.

Although primarily been examined in the context of red flowers, niche segregation between flowers pollinated by bees (hereafter bee‐flowers) and birds (hereafter bird‐flowers) can also occur in other parts of the colour spectrum (Papiorek *et al*. 2016; Camargo *et al*. 2019; Coimbra *et al*. 2020). In the case of white flowers, lack of UV reflection enhances spectral purity and contrast for bees, while white UV‐reflective flowers provide lower purity and contrast for bees (Lunau *et al*. 2011; Coimbra *et al*. 2020). In accordance with the bee‐avoidance hypothesis, white bee‐flowers often absorb UV, while white bird‐flowers often reflect UV (Lunau *et al*. 2011; Camargo *et al*. 2019). However, it is important to note that UV reflection in white flowers may vary depending on ecological context and background contrast, and some bee‐pollinated species exhibit UV‐reflective white flowers (Lunau *et al*. 2021). Such exceptions may reflect local adaptation, background conditions, or variation in floral signal efficiency across environments. Yellow flowers present a different case, characterized by UV patterns where petal tips reflect UV while central areas absorb it, serving as visual guides to floral resources and landing sites for bees (Lunau 1992, 1993; Lehrer *et al*. 1995; Lunau *et al*. 1996; Papiorek *et al*. 2016; Scaccabarozzi *et al*. 2023). Yellow bee‐flowers often present UV colour patterns, while yellow bird‐flowers do not exhibit such patterns and this lack of visual guides can discourage visitation by bees (Papiorek *et al*. 2016). Consequently, UV reflectance emerges as a key colour property mediating attraction and exclusion processes in bee‐ versus bird‐flowers.

There are several evolutionary shifts from bee to bird pollination that are accompanied by changes in flower colour (Barreto *et al*. 2024). Such transitions have been documented in specific lineages within genera such as *Aquilegia* (Whittall & Hodges 2007), *Costus* (Kay & Grossenbacher 2022), *Penstemon* (Castellanos *et al*. 2004), and *Salvia* (Wester *et al*. 2020), where floral traits including colour have diverged in association with pollinator shifts. These examples highlight the importance of considering the evolutionary transitions of flower colour and the complexity of interpreting floral colour signals from different visual systems, including those of effective pollinators and floral antagonists (Rodríguez‐Gironés & Santamaría 2004; Rausher 2008).

Patterns across pollination systems suggest that shifts from bee to bird pollination are commonly associated with changes in floral traits involved in signal detection—not only in hue, but also in UV reflection (Lunau *et al*. 2011; Camargo *et al*. 2019; Coimbra *et al*. 2020). Therefore, floral UV reflection is likely to be shaped by the strength of selective pressures exerted by pollinators and antagonists, through mechanisms involving both attraction and sensory exclusion. Our study addresses this relationship by combining phylogenetic comparative methods with models of trait evolution, providing novel insights into the interplay between floral colour evolution and pollinator‐mediated selective pressures. Bee‐ and bird‐flowers serve as excellent models for studying the evolution of floral UV reflection, because the bee‐avoidance hypothesis predicts different selective pressures in floral UV in each of these plant groups (Lunau *et al*. 2011; Papiorek *et al*. 2016). Furthermore, bees and birds are the most prominent pollinator groups among insects and vertebrates, respectively, and bee‐ and bird‐flowers have evolved several times across the angiosperm phylogeny (Ollerton *et al*. 2011; Stephens *et al*. 2023).

Specifically, we characterized the UV reflection and human hue categories (red, white, and yellow) of the flowers to investigate whether UV reflection and floral colour are evolutionary labile or constrained by shared ancestry. Moreover, we explore whether the evolution of UV reflection reflects adaptations to the specific type of pollinator by employing different adjusted Brownian motion (BM) and Ornstein‐Uhlenbeck (OU) models of trait evolution. BM models simulate trait evolution under a neutral process with random drift, while OU models, that have been successfully applied to floral traits, incorporate stabilizing selection towards adaptive optima (Smith & Donoghue 2008). These models allow the estimation of evolutionary parameters (adaptative optima, evolutionary rate of change, and selective strength) that help us to better interpret the evolutionary trajectories of a trait.

**Fig. 1:**
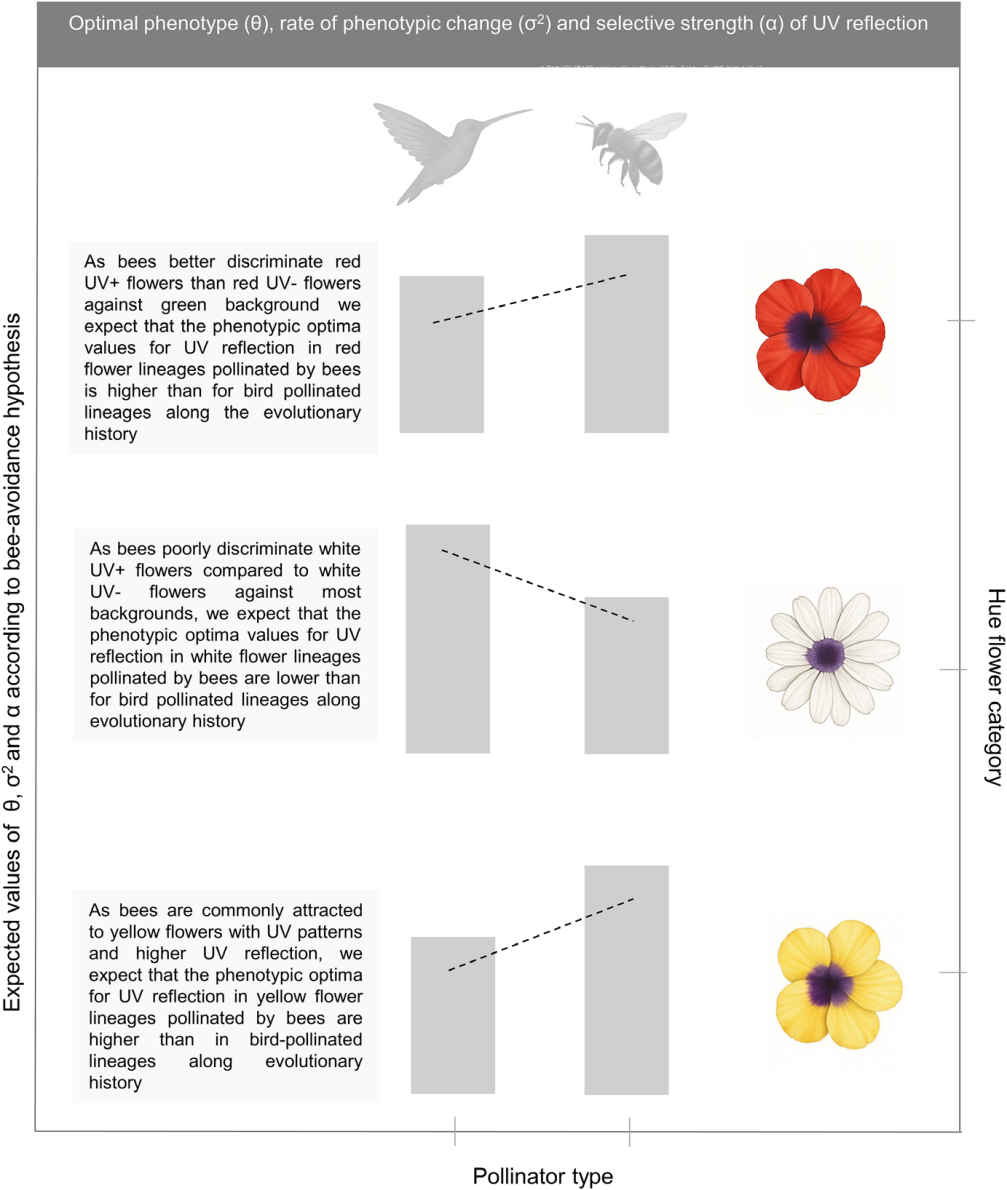
The expected evolutionary patterns of floral UV reflection for each association between hue category (red, white, and yellow) and pollinator type (bees vs. birds). These expectations are based on pollinator attraction and sensory exclusion processes. The flower hue category (red, white, yellow) is shown for both pollinator types, highlighting the expected differences in the adaptative optima (θ), evolutionary rate of change (σ^2^), and selective strength (α) of UV reflection. The highlighted boxes on the left provide explanations for the expected values of evolutive parameters in each scenario of flower hue category versus pollinator type. Pollinators vector sources: Freepik Company S.L. version: 15 April 2024. freepik.com.

We tested if bee‐ and bird‐pollinated red, white, and yellow flowers diverge in their adaptive optima, evolutionary rates and selective strengths of UV reflection. Following the bee‐avoidance hypothesis, we predict that the adaptive optima, evolutionary rate, and selective strength of UV reflection in red and in yellow bird‐flower lineages are lower than for red and yellow bee‐flower lineages. On the other hand, the adaptive optima, evolutionary rate and selective strength of UV reflection in white bird‐flower lineages should be higher when compared to white bee‐flower lineages (Fig. 1).

## MATERIAL AND METHODS

### Dataset

The species included in this study are part of a curated dataset that includes floral spectral reflectance and colour hue, floral visitors, and the categorized pollination systems for 245 angiosperm species (Oliveira *et al*. unpubl data). Among these, the reflectance of 204 species has not yet been published, significantly enhancing our knowledge of floral colour. Part of the spectral reflectance data was measured *in situ* by two of the co‐authors (K. Lunau and S. Gerten) during several field trips in distinct native vegetations and botanical gardens. We complemented this effort with the datasets of Coimbra *et al*. (2020).

The species are spread across 180 genera in 71 families. The most representative families in number of species were Asphodelaceae, Asteraceae, Bromeliaceae, and Fabaceae, which are mostly distributed across the Neotropics (Table S1). The species are distributed along an altitudinal range from −300 (records on Palestine, near the Dead Sea) to 4,669 m a.s.l. The temperature and annual precipitation levels across their distribution range from −2.1 to 29.7°C and 4 to 5446 mm, respectively. Annual UV‐B irradiation levels range from 3019 to 9,979,344,482 J m^−2^ (Hijmans *et al*. 2005; Beckmann *et al*. 2014).

We focused our analysis on species with clear pollination syndromes dominated either by bees or by birds. Although our database includes broader records of floral visitors – including antagonists and occasional pollinators – only species with a predominant and well‐documented pollinator group were included in the analyses. A more detailed description of the criteria for classifying pollinators is provided in “Pollinators” section. The role of floral antagonists is discussed in the context of floral trait evolution in the “Discussion” section.

### Spectral reflectance

We restricted the reflectance data to the 300–700 nm wavelength range, which falls within the spectral sensitivity of most flower visitors (Chittka & Raine 2006; Kelber 2019; Van Der Kooi *et al*. 2021). The *in situ* measurements were acquired using a surface spectrophotometer (USB 2000, USB 4000; Ocean Optics) coupled with a probe at 45° to the measuring spot, calibrated with white barium sulfate (BaSO_4_) as white standard and no light as a black standard (Lunau *et al*. 2011). For each of the 245 species, two to five reflectance measurement was taken from one or a few individuals, depending on field availability. Measurements were taken from the most visually conspicuous floral structures – including petals, sepals, stamens, and bracts – and were averaged to produce a single mean reflectance curve per species. To ensure consistency, we prioritized structures that are visually conspicuous to human eyes at long distances, as these are more likely to influence initial pollinator attraction (Table S1; Menzel *et al*. 1997; Spaethe *et al*. 2001). For the sake of simplicity, we refer to all measurements as “flowers”.

To detect possible UV patterning, we followed the protocol described in Papiorek *et al*. (2016), in which each flower was separated into two regions: the center (including ray florets, corolla orifices, basal parts of petals or flags, and reproductive organs, i.e., areas where nectar guides typically occur) and the periphery (disc florets, lips, adaxial parts of corollas, and peripheral parts of petals regions). The spectrophotometer probe was relocated across these regions to check and control cases of variation in spectral reflectance. When UV patterning was detected – most often in yellow flowers but also in other colours – we measured both UV‐reflecting and UV‐absorbing regions and calculated the arithmetic mean of their reflectance curves to obtain an overall floral signal. This approach was applied uniformly, regardless of flower colour. Importantly, such cases were less frequent than those without any detectable UV signal variation across the floral structures measured. Our aim was to summarize UV reflectance at the whole‐flower level in line with our broader comparative analyses across species, rather than to model spatial variation in UV patterns. While spatially explicit analyses were beyond the scope of this study, we acknowledge that such patterns can be important for pollinator behaviour and may represent a promising avenue for future research. For species with flowers exhibiting multiple colours, we chose the reflectance of the colour occupying the more significant area (Spaethe *et al*. 2001).

### Hue categories and UV reflection

We categorized the floral colour hues of each species based on their average reflectance (+) or absorbance (−) in the UV (u: 300–400 nm), blue (b: 401–500 nm), green (g: 501–600 nm), and red (r: 601–700 nm) range of wavelengths. To differentiate reflectance from absorbance in each waveband, we applied empirically derived thresholds of 10% for UV, 30% for blue, 40% for green, and 60% for red reflectance, following Camargo *et al*. (2019) and Coimbra *et al*. (2020). These thresholds correspond to percentage reflectance values and are used to identify prominent spectral peaks indicative of colour expression in each range. A “+” indicates that a flower reflects a substantial proportion of light in that band, while a “−” indicates low reflectance, or relative absorption. Flowers whose reflectance in the green band differed by 50% or more from the average reflectance in the blue and red bands were categorized as green‐absorbing (g−) (Camargo *et al*. 2019; Coimbra *et al*. 2020; Table S1). These thresholds were originally proposed for distinguishing major spectral patterns relevant to pollinator perception and are supported by evidence of their effectiveness in differentiating visual signals under bee and bird visual systems.

We acknowledge that summarizing spectral data into mean reflectance values, while effective for broad comparisons, does not capture the full shape of the spectral curve – which can influence how colours are perceived by different pollinators. Still, our thresholds follow previous ecological applications and were supported by additional validation analyses. We examined how our hue and UV reflection categories relate to chromatic contrast under bee and bird visual systems (applying a standard green background to simulate a generalized ground vegetative) and found that our categorization recovers biologically meaningful groups (see Tables S4–S6). This choice allows consistent comparisons across species, as most floral signals are displayed against leaves, stems, or ground vegetation. However, we recognize that this approach simplifies background variability, which is likely to differ among habitats and studies from which the reflectance data were obtained. Nevertheless, this approach allowed us to perform standardized comparisons across a large number of species and clades, which would not be feasible with more complex perceptual modelling. Future work could refine this framework by incorporating full spectral profiles and explicit models of pollinator vision.

Based on this classification, we assigned each species to one of the following abundances and hue categories: 2 cyan (u−b+g+r− or u+b+g+r−), 3 green (u−b−g+r− or u+b−g+r−), 3 black (u−b−g−r− or u+b−g−r−), 24 blue (u−b+g−r− or u+b+g−r−), 46 pink (u−b+g−r+ or u+b+g−r+), 47 yellow (u−b−g+r+ or u+b−g+r+), 75 white (u−b+g+r+ or u+b+g+r+), and 94 red (u−b−g−r+ or u+b−g−r+). In this notation, each waveband is represented by a plus (+) or minus (−) sign to indicate high or low reflectance, respectively, relative to the empirically defined thresholds described above. For example, red flowers were characterized by high reflectance in the red band (>60%) and low reflectance in other bands, while white flowers showed high reflectance across all visible bands. We further calculated UV reflection as the proportion of UV reflectance (300–400 nm) relative to total reflectance (300–700 nm). We acknowledge that absolute reflectance values may vary due to instrument sensitivity, particularly near spectral extremes, but our sampling and calibration protocols (see below) minimized measurement noise. Our classification represents the reflectance patterns that are typical of UV and non‐UV flowers of each of these hue categories (Fig. S1).

To better capture the ecological and perceptual relevance of floral UV reflection under distinct pollinator visual systems, we grouped all species into two distinct datasets: one comprising flowers categorized as white, red, or other colours (hereafter referred to as the “white‐red‐flowers set”) and another consisting of flowers categorized as yellow or other colours (hereafter the “yellow‐flowers set”) (Fig. S2). This division reflects known differences in how bee and bird visual systems process colour and UV signals. White and red UV‐reflecting or UV‐absorbing flowers are processed similarly in both visual systems, as the presence or absence of UV reflection determines whether chromatic or achromatic channels are used to discriminate these colours (Lunau *et al*. 2011). In contrast, yellow UV‐reflecting or UV‐absorbing flowers are chromatic to both bees and birds. In this case, it is the presence of UV patterns that ultimately increases chromatic contrast for bees, rather than purely chromatic or achromatic signals (Papiorek *et al*. 2016). By analysing these groups separately, we aimed to account for their distinct visual ecologies and maximize the resolution in detecting differences in UV reflection evolution that are linked to pollinator type. This approach allows us to test whether floral UV reflection evolved under different selective pressures in bee‐ versus bird‐pollinated flowers of different hues.

### Pollinators

We categorized each plant species as bee‐ or bird‐pollinated (hereafter, bee‐flowers and bird‐flowers) based on the primary pollination system reported in the literature. We followed the functional guild framework of Ollerton *et al*. (2007), which classifies pollinators according to their ecological roles rather than strict taxonomic identity. A list of pollinator references is provided in Table S1. We compiled pollinator data from Google Scholar and ISI Web of Science databases using keywords such as “species name“*”pollination”, “pollinator” or “species name“*”floral visitors.” We also included data from theses, dissertations, floras, and regional ecological studies whenever detailed observations of visitation frequency and pollination effectiveness were available. Each species was assigned to a single dominant pollinator category (bees or birds) based on the consensus from published sources or, when conflicting information existed, on the most frequently reported effective pollinator. While our analyses focus on primary pollinators, we acknowledge that many flowers interact with a broader community of floral visitors, including inefficient pollinators and antagonists. This broader context is relevant for interpreting the potential role of UV reflection not only in attraction but also in the exclusion of less effective visitors, as predicted by the bee‐avoidance hypothesis. In total, we identified 136 species as bee‐flowers and 109 as bird‐flowers.

### Phylogeny

Inferences about the phylogenetic relationships of angiosperms were based on the time‐calibrated phylogeny ALLMB (Smith & Brown 2018), which included the largest number of species sampled here. This phylogeny combines data from GenBank and from the Open Tree of Life, with the backbone of Magallón *et al*. (2015). To include as many species as possible, we assumed that distances within the same genus or between sister genera are smaller than those at other hierarchical levels, and we corrected for missing species in the phylogeny by substituting them with species from the same genus if present in the tree, or with species from sister genera if absent. This procedure creates polytomies by adding absent genera or species to their closest taxa, which were then automatically solved using the *multi2di* function of the ape package (Paradis *et al*. 2004; Paradis & Schliep 2019).

### Ancestral reconstruction

We conducted ancestral reconstruction of hue categories and UV reflection for each of the two sets (white‐red‐flowers and yellow‐flowers) to infer the evolutionary history of these traits along the phylogeny and determine whether evolution of all hue categories was labile or conserved. To reconstruct hue categories, we fitted different evolutionary models since we had no prior hypothesis of the evolution of hue categories (Harmon *et al*. 2008). We selected among the equal rates (ER), all different rates (ARD) and symmetrical rates (SYM) models by comparing Akaike weights and ∆AICc, and used the best‐fitted model (with ∆AICc ≤2) to perform a stochastic character mapping with 1000 simulations to evaluate the robustness of the generated reconstruction (Revell 2012). In the ER model, an equal transition rate is assumed among state pairs, while in the ARD model, different transition rates are allowed. The SYM model indicates that the transition rate from one state to another is the same. However, distinct pairs of trait states can still have different overall transition rates. We also investigated the reconstruction of UV reflection based on the Brownian motion model and estimates of internal node states using maximum likelihood and interpolated each edge state (Felsenstein 1985; Revell 2012).

### Trait evolution

To explore if the evolution of UV reflection in white, red, and yellow flowers was associated with pollinator types (bee vs. bird), we built Brownian motion (BM) and Ornstein–Uhlenbeck (OU) models of trait evolution (Hansen 1997; O'Meara *et al*. 2006; Beaulieu *et al*. 2012). The BM model assumes that traits evolve randomly or under stabilizing selection following a changing optima value, while the OU model assume that the evolution of traits occurs under a selective regime. The OU model estimates the adaptative optima (θ) towards which populations evolve in an OU process, the evolutionary rate of change (σ^2^), i.e., the level of variation around the optima along the evolutionary process, and selective strength (α), i.e., the magnitude of change in the trait towards the optima (Hansen 1997; Beaulieu *et al*. 2012). We fitted seven models of UV reflection evolution for each of the two sets of flowers following the model‐fitting framework of de Alencar *et al*. (2017): (1) BM1‐ BM model of single‐rate, (2) OU1‐ OU model of single optimum, (3) BMS‐ multi‐rate BM model allowing different σ^2^ values for each association between hue and pollinator categories, (4) OUM‐ OU model assuming different θ values and single σ^2^ and α values for each association between hue and pollinator categories, (5) OUMV‐ OU model allowing distinct θ and σ^2^ values for each association between hue and pollinator categories, (6) OUMA‐ OU model allowing different θ and α values for each association between hue and pollinator categories and (7) OUMVA‐ OU model that assumes different θ, σ^2^, and α values for association between hue and pollinator categories. We also fitted simmap trees estimated under the associations between hue and pollinator categories (Beaulieu & O'Meara 2016) and calculated ΔAICc to investigate which model best explained UV reflection evolution in each simmap tree simulated. The best fit models were those with ΔAICc less than two (Burnham & Anderson 2004).

According to the selection of the best‐fit model, we employed Generalized Linear Mixed Models (GLMM) using the UV‐reflectance optima values (θ) as response variable. We used as explanatory variables the pollinator type (bee or bird), the hue category (white, red or others), as well as the interaction between these two terms. We considered the simulation as a random factor. We used the Gaussian family, after checking model assumptions via residual diagnostics, including tests for uniformity, dispersion, and identification of outliers, alongside visual inspections of residual plots. Visual examinations indicated that the residuals behaved reasonably well. However, the Kolmogorov–Smirnov test results suggest that the model may not fully capture the underlying data structure. This discrepancy was likely due to the presence of outliers, which can significantly influence both the mean and variance of the data. Despite this, we found that the Gaussian family provided the best fit for the model after comparing the adjustment of the model under distinct distributions and predictor transformations (Figs. S5 and S6). We also used Estimated Marginal Means (*emmeans*) analysis to explore the estimated marginal means for the combinations of pollinator type and hue categories. As a post‐hoc test, we assessed the significance of differences between these combinations, employing *P*‐values adjusted for multiple comparisons using the Tukey correction. We conducted the same approach for the yellow flowers set, using Gaussian family distribution. In this case, hue included yellow versus other colour categories.

### Data analysis packages

We used the R software environment, v. 4.2.1 (R Core Team 2023) to conduct all analyses. We used pavo (Maia *et al*. 2013, 2019) and forcats (Wickham 2023) packages to explore spectral properties of colours, calculate the chromatic and achromatic contrasts, and conduct hue categorization and manipulation of categorical factors. Regarding phylogenetic reconstruction of continuous and discrete traits, we employed a combination of phytools (Revell 2012) and geiger (Harmon *et al*. 2008) packages. The ape (Paradis *et al*. 2004; Paradis & Schliep 2019), ggplot2 (Wickham *et al*. 2016), and ggtree (Yu *et al*. 2017) packages were used to import and manipulate phylogenetic trees, correct polytomies, and results visualization. In addition, we used OUwie package (Beaulieu & O'Meara 2016) to fit evolutionary models, and the packages glmmTMB (Magnusson *et al*. 2017), car (Fox *et al*. 2019), DHARMa (Hartig 2018), and *emmeans* (Lenth 2022) to fit the GLMMs, perform model diagnostics, and estimate marginal means.

## RESULTS

### Hue categories and UV reflection

We present results of our comparative analyses on UV reflectance and floral colour composition across the studied species. Within the white‐red flowers set, 94 species (32.5%) were categorized as “red”, 74 species (25.4%) as “white” and 124 species (42.5%) as “other”. In the yellow‐flowers set, the “yellow” category comprised 46 species (16%), while the “other” category encompassed 246 species (84%) (Table S1).

The observed UV reflection values varied extensively across the studied species (Table S1, Figs. S1 and S2). On the lower end of the spectrum, the recorded minimum UV reflection was 0.000001 (*Brownea ariza* Benth. ‐ Fabaceae and *Vriesea neoglutinosa* Mez ‐ Bromeliaceae). Conversely, at the higher end, the maximum value was 0.77 (*Heliconia bihai* (L.) L. ‐ Heliconiaceae). The mean UV reflection across all species was 0.09 ± 0.11. Notably, 197 species (67.5%) displayed a UV reflection below 0.1 (UV−), while 93 species (31.8%) exhibited a UV reflection exceeding 0.1 (UV+; Table S1).

### Ancestral state reconstruction

**Fig. 2:**
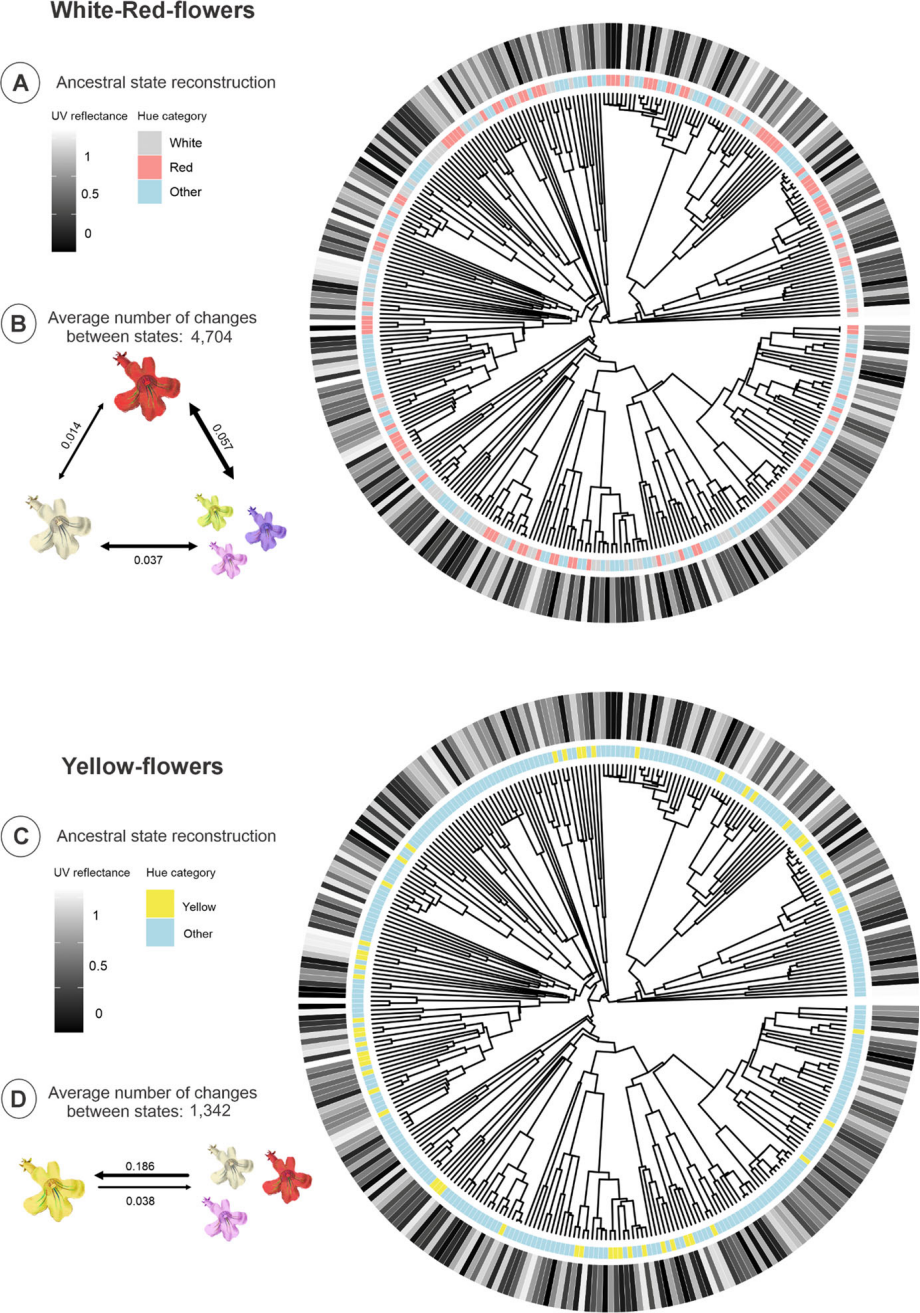
(A) Ancestral state reconstruction of hue categories and UV reflection for the white‐red‐flowers set. (B) Transition rates among the hue category states (white, red, and other colours) in the SYM (symmetrical rates) model. (C) Ancestral state reconstruction of hue categories and UV reflection for the yellow‐flowers set. (D) Transition rates among the hue category states (yellow and other colours) in the ARD (all different rates) model. The thickness of the arrows indicates the frequency of change between states. Both analyses were conducted considering 245 species spread across 205 genera in 76 families. The tree is dated in millions of years (Ma). Flower image sources: Watson, L., and Dallwitz, M.J. 1992 onwards. The families of flowering plants: descriptions, illustrations, identification, and information retrieval. Version: 15 April 2024. delta‐intkey.com.

Among the white‐red flowers set, ARD and SYM models provided the best fit (Table S2). Despite the ARD model displayed a slightly lower AICc, the differences between SYM and ARD were minimal, and the SYM model was used for subsequent analyses as it is the simpler model. Ancestral state reconstructions using UV reflection revealed a high frequency in state changes (UV− to UV+ and vice‐versa; Fig. 2a–c). The rate of transition between red and other hue states was higher than transitions involving the white hue. Additionally, the rate of transition between white and other hues was greater than between white and red hue states. On average, there were at least approximately 4704 transitions between hue states (Fig. 2b). In yellow‐flowers, the ARD model provided the best fit (Table S3). On average, the analysis estimated that there were at least 1342 changes between yellow and other hue states (Fig. 2d). Taken together, these results show that the evolution of colour traits is highly labile (Fig. 2).

### Trait evolution

For the white‐red‐flowers set, the OUMV (Ornstein‐Uhlenbeck model with different optima and rates) provided the best fit across most stochastic mappings (68.3% of total fits), followed by the OU1 model (38.9% of total fits; Table 1). The OUMA (Ornstein‐Uhlenbeck model with different optima and selection strengths) provided the best fit in the case of the yellow‐flowers set (74.5% of total fits), followed by OU1 (22.8% of total fits; Table 1).

**Table 1:** Number of model fits in the analyses of UV reflection evolution in the white‐red‐flowers set and in the yellow‐flowers set.

set	model	best fit	ties						
			BM	BMS	OU1	OUM	OUMA	OUMV	OUMVA
White‐Red flowers	BM	0	‐						
	BMS	0	0	‐					
	OU1	378	0	0	‐				
	OUM	5	0	0	5	‐			
	OUMA	8	0	0	0	0	‐		
	OUMV	663	0	0	89	0	0	‐	
	OUMVA	111	0	0	0	0	0	0	‐
Yellow flowers	BM	0	‐						
	BMS	0	0	‐					
	OU1	209	0	0	‐				
	OUM	26	0	0	18	‐			
	OUMA	682	0	0	113	12	‐		
	OUMV	40	0	0	0	0	0	‐	
	OUMVA	94	0	0	1	0	2	0	‐

Ties represent runs in which two models exhibited ΔAICc <2 relative to the adjusted simulated sample of simmaps fitted for each model separately. The best model frequency in each row does not sum the number of fittings of each reference model (1000) because some simmaps did not suggest a best model. In total, 971 simmaps were analysed out of 1000 simmaps simulated for the white‐red flowers, while 915 for yellow‐flowers. Among these, the OUMV model showed best fit in 663 cases for the white‐red‐flowers, and the OUMA showed best fit 682 times for the yellow‐flowers set (highlighted in grey).

**Fig. 3:**
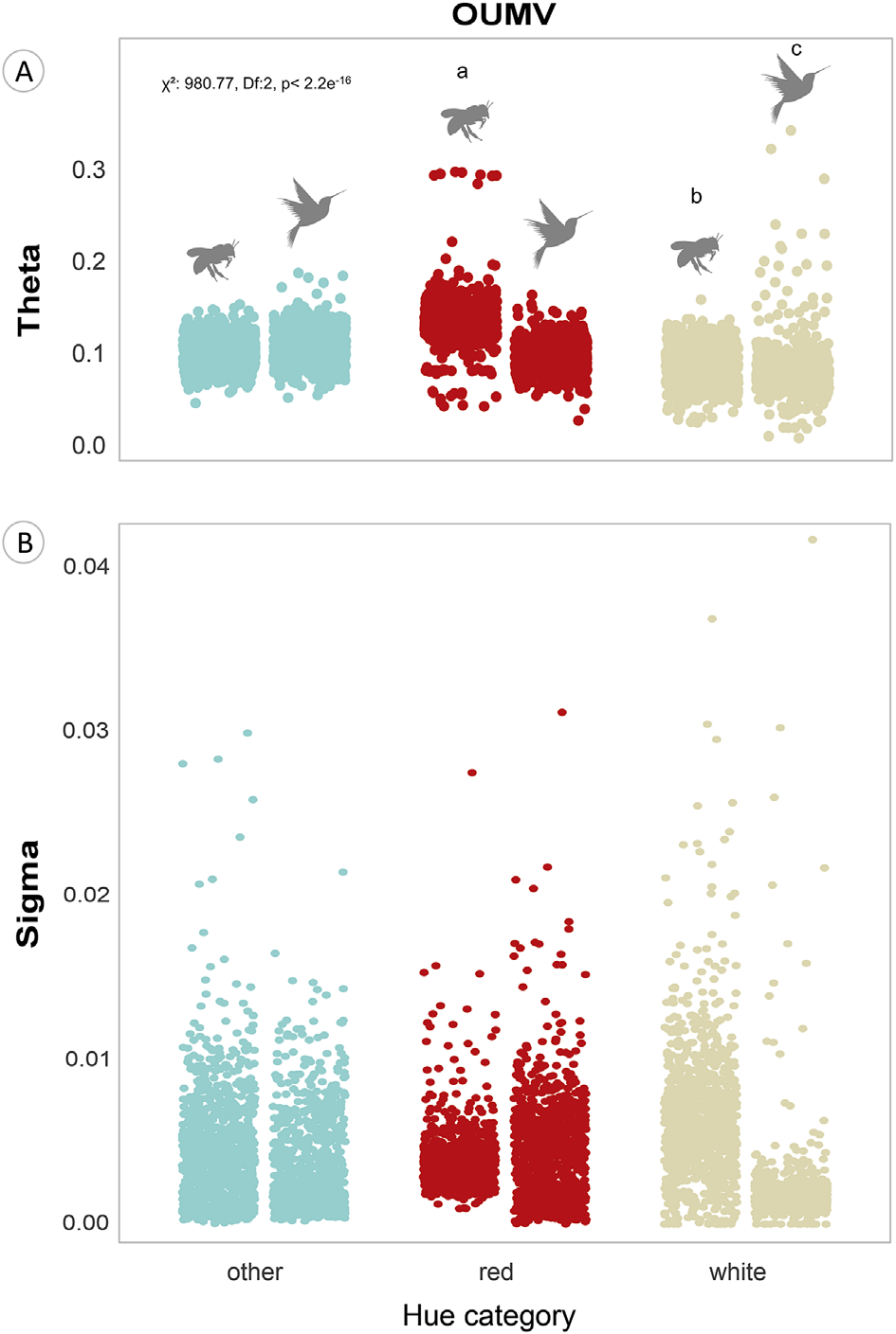
Estimates of parameters from the OUMV model, selected as the best model explaining the evolution of UV reflection in the white‐red‐flowers subset. (A) Adaptive optima (θ ‐ theta; % UV reflection) for each association between hue category and pollinator type within this group of flowers. Letters above categories represent groupings from Tukey post‐hoc comparisons across all hue × pollinator combinations. Specific comparisons highlighted in the main text (e.g., red bee‐flowers vs. red bird‐flowers) are detailed in Table S7. Statistical significance of the interaction term: *χ* ^2^ = 980.77, df = 2, *P* < 0.001. (B) Evolutionary rates of change (σ^2^ – sigma); (% UV reflection) ^2^ per million years (Ma) around the optima for each association between hue categories and pollinator types. Pollinators vector sources: Freepik Company S.L. Version: 15 April 2024. freepik.com.

There were differences in UV reflection optima (θ) and evolutionary rates of change (σ^2^) between hue and pollinator associations in the white‐red‐flowers set. Higher values of θ were estimated for lineages of red and white bee‐flowers compared to their bird‐flowers counterparts (Δθ = 0.038, *P* < 0.001 for red, Δθ = 0.0088, *P* < 0.001 for white; Fig. 3a, Table S7). A similar trend for σ^2^ was observed (Fig. 3b), although no formal statistical test was performed for this parameter. Interestingly, white bee‐ and bird‐flowers exhibited a small difference in UV reflection optima (Δθ = 0.0074, *P* < 0.001; Table S7). Notably, white bird‐flowers displayed a substantially higher variation in optima values, including the highest values in the dataset compared to their bee‐flowers counterparts (Fig. 3a). Moreover, white bee‐flowers also exhibited greater visual variability around the evolutionary rate of change (σ^2^) compared to white bird‐flowers (Fig. 3b). UV reflection optima depended on the interaction between pollinators and hue category (*χ* ^2^: 980.77, df: 2, *P* < 0.01; Table S7). We identified the highest UV reflection optima in red bee‐flowers (θ: 0.038, *P* < 0.01), followed by white bee‐flowers (θ: 0.008, *P* < 0.01) and flowers of other colours pollinated by birds (θ:0.004, *P* < 0.001) when compared with optima from the same hue categories but with the opposite pollinator (Table S7).

**Fig. 4:**
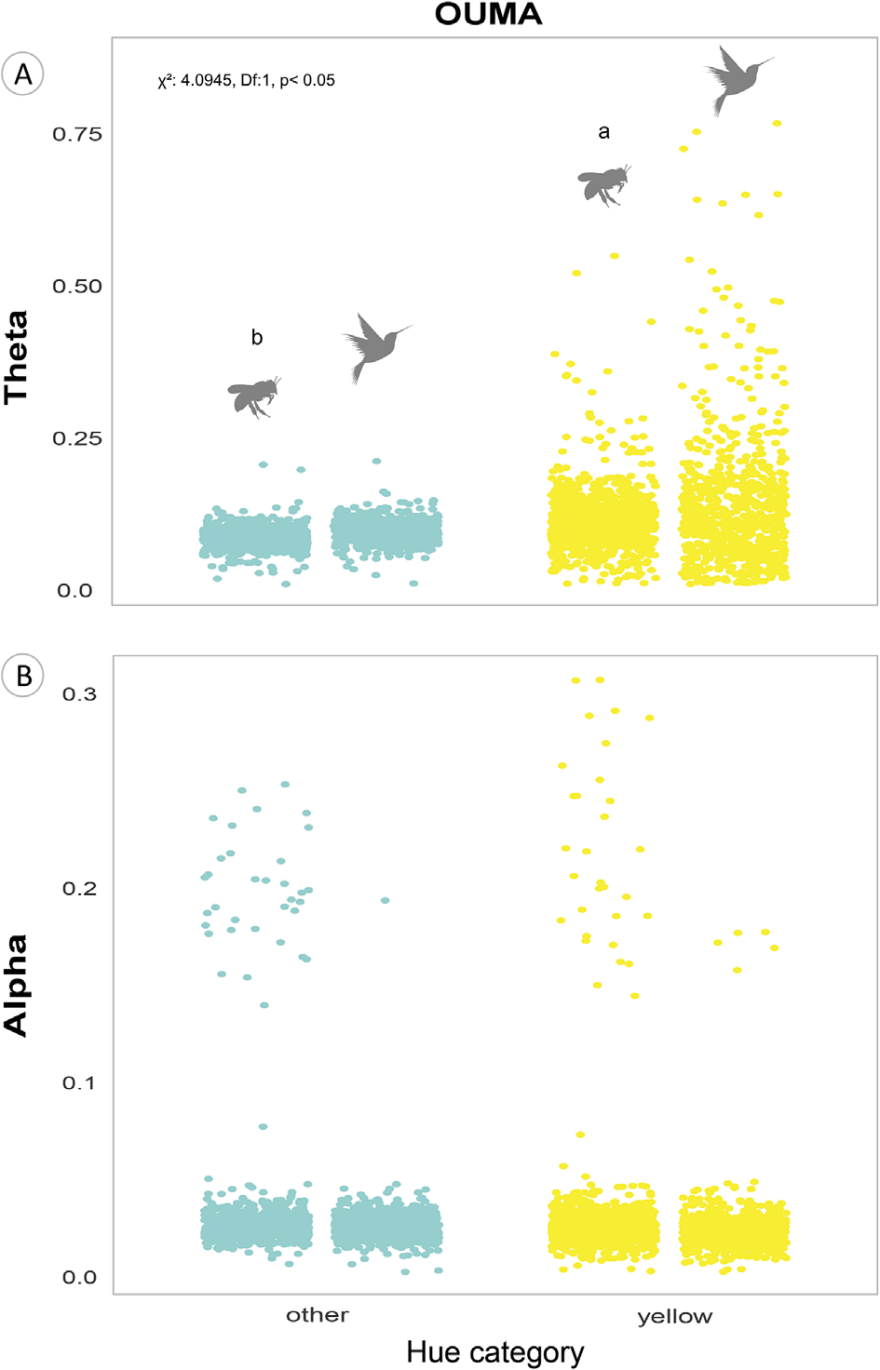
Estimates of parameters derived from the OUMA model, selected as the best model explaining the evolution of UV reflection in the yellow‐flowers subset. (A) Adaptive optima (θ ‐ theta; % UV reflection) for each association between hue category and pollinator type within this flower group. Letters above categories represent groupings from Tukey post‐hoc comparisons across all hue × pollinator combinations. Specific comparisons highlighted in the main text (e.g., yellow bee‐ vs. yellow bird‐flowers) are detailed in Table S8. Statistical significance of the interaction term: *χ* ^2^ = 4.0945, df = 1, *P* < 0.05. (B) Selective strength (α ‐ alpha; Ma^−1^) around the optima for each association between hue categories and pollinator types. Pollinator vector sources: Freepik Company S.L. Version: 15 April 2024. freepik.com.

In the yellow‐flowers set, we also found differences in UV reflection optima (θ) and the selective strength (α) between hue and pollinator associations. Yellow bee‐flowers tended to exhibit lower optima values (θ) than yellow bird‐flowers (Δθ = 0.0041, *P* = 0.282; Table S8; Fig. 4a). Yellow flowers consistently exhibited higher optima in comparison with the other hue categories, highlighting their unique adaptive strategies independently of pollinator type (Table S8). Moreover, yellow and other bee‐flowers visually displayed higher selective strength (α) than bird‐flowers of the same hue categories (Fig. 4b). This suggests a faster rate of evolutionary change in UV reflection towards the optimum value among bee‐pollinated flowers. The interaction between pollinator and hue category significantly affected the optima values in the yellow‐flowers set (*χ* ^2^: 4.0945, df: 1, *P* < 0.05). Nevertheless, most comparisons between pollinator and hue categories did not show significant differences, with the exception of yellow bee‐flowers, which had significantly higher optima than other bee‐flowers (Δθ = 0.007; *P* = 0.0133; Table S8).

## DISCUSSION

Our study revealed a wide variation in floral UV reflection in bee‐ and bird‐flowers, an important colour trait that mediates pollinator attraction and exclusion of less effective flower visitors. Ancestral state reconstructions showed that hue and UV reflection displayed multiple transitions, suggesting the labile nature of these colour traits. Bee‐ and bird‐flowers exhibited hues and UV reflection states that produced higher chromatic contrast in the visual system of their corresponding pollinators and lower contrast for less effective ones, demonstrating the link between colour processing and the evolution of floral colours. The correspondence between chromatic contrast and flower visitors, aligned with the estimated evolutionary parameters, underscores the diversity of floral signalling strategies related to the visual capabilities of pollinators and antagonists. Nevertheless, the trait evolution models showed that the evolutionary responses varied between red, white, and yellow flowers. Thus, pollinator attraction and exclusion of less effective flower visitors acted differently in each of these groups of flowers. Below, we further discuss the potential mechanisms behind these evolutionary patterns.

We observed a wide range of floral UV reflection (proportion relative to total reflection) across species (Fig. S1). Most species (67.46%) exhibited an average UV reflection below 0.1, indicating that the other one‐third of species reflects UV. This proportion is quite significant considering that floral UV reflection is often lower and less frequent in comparison with other ranges of the spectrum (Chittka *et al*. 1994; Tunes *et al*. 2021). This relatively high proportion of UV‐reflecting flowers is likely related to the subset of studied plants, i.e., bee‐ and bird‐flowers. Thus, UV reflection may be more common than expected when considering certain groups of pollinators. It is also important to acknowledge that factors such as UV exposure, temperature, and precipitation also play an important role influencing floral UV reflection (Koski & Ashman 2016; Dalrymple *et al*. 2020; Koski *et al*. 2020). UV‐absorption in the central parts of the flowers also protects against UV radiation‐induced damage to plant reproductive structures (Koski & Ashman 2014, 2015b; Brock *et al*. 2016; Papiorek *et al*. 2016). Therefore, the interplay between UV reflection and absorption, their functions in attracting pollinators, and their protective role significantly contributes to the diversity and distribution of flower colours among plant species.

The scattered distribution of different hue categories and UV reflection across the angiosperm phylogeny indicates a labile evolution of these floral traits. Indeed, several studies on floral reflectance showed that closely related species do not exhibit similar floral colour properties, such as hue and saturation (Muchhala *et al*. 2014; Shrestha *et al*. 2014; Gómez *et al*. 2015; Bergamo *et al*. 2018). However, depending on the clade, UV reflection can either be constrained by evolutionary relatedness (Koski & Ashman 2016; Tunes *et al*. 2021), or exhibit high variation among closely related species (Rieseberg & Schilling 1985; Naruhashi 1999; Koski & Ashman 2016). Here, we investigated the evolution of UV reflection at a broader phylogenetic scale encompassing the angiosperms, using a relatively small sample for which reflectance and pollinator data were available. Although our sample includes a limited number of species relative to the overall diversity of angiosperms, it spans a wide range of lineages and pollination systems. As such, it provides a valuable initial framework to explore macroevolutionary patterns of UV reflection. We acknowledge that broader taxon sampling could enhance the resolution of evolutionary inferences, and we encourage future studies to expand upon these findings within more densely sampled clades. Even with limited sampling, our results indicate that UV‐reflection and UV‐absorption have evolved repeatedly across diverse angiosperm lineages. Although our dataset does not allow a detailed reconstruction of all ancestral states or transitions, these patterns highlight promising directions for future studies within more densely sampled clades (Rausher 2008).

The macroevolutionary models and GLMMs revealed a remarkable pattern of floral UV reflection evolution, highlighting the pivotal role played by pollinators and less effective flower visitors. Red bee‐flowers showed higher UV reflection optima (θ) than red bird‐flowers. This is in accordance with the bee‐avoidance hypothesis, which states that sensorial exclusion of bees drove the evolution of less contrasting bird‐pollinated red flowers (Lunau *et al*. 2011; Bergamo *et al*. 2016). Although the number of red bee‐pollinated species was relatively small, the observed pattern of higher UV reflection optima remained consistent across model iterations and was supported by statistically significant contrasts. This suggests that, even with a limited sample, red bee‐flowers tend to exhibit distinct UV reflection strategies consistent with the bee‐avoidance hypothesis. Future studies with more targeted sampling could further test these associations in detail.

Bees focus their foraging efforts on red flowers with a secondary reflectance peak in the UV band, which are more chromatic to bees (Lunau *et al*. 2011; Chen, Niu, *et al*. 2020). Accordingly, we found that bee‐flowers exhibit greater chromatic contrast in the visual systems of bees compared to bird‐flowers, whose visual system is particularly sensitive to chromatic contrasts (Spaethe *et al*. 2001; Van Der Kooi *et al*. 2019). In contrast, flower colours that are inconspicuous for bees might be attractive for birds, which effectively exploit a specialized niche of red flowers that absorb UV light (Lunau *et al*. 2011; Bergamo *et al*. 2016; Camargo *et al*. 2019). Importantly, red bird‐flowers also exhibited high chromatic contrast in the bird visual systems, suggesting that both bird attraction of birds and bee exclusion may have influenced the evolution of red flowers (Rodríguez‐Gironés & Santamaría 2004; Shrestha *et al*. 2013).

We also found a slightly higher UV reflection optima in white bee‐flowers compared to white bird‐flowers. This suggests that the difficulty bees face in distinguishing white UV‐reflecting flowers against green foliage (Chittka 1992; Chittka & Menzel 1992; Kevan *et al*. 1996), which may discourage visitation (Kevan *et al*. 1996; Lunau *et al*. 2011; Camargo *et al*. 2019), does not impose as strong a selective pressure as observed in red flowers. However, it is noticeable that white bird‐flowers displayed greater dispersion around their optima, presenting the highest optima values (see Fig. 3a). Thus, sensory exclusion pressures resulting in high UV reflection optimum may have occurred in specific white bird‐pollinated flower lineages. In this context, white bird‐flowers also exhibited low chromatic contrast in the bee visual system. Their higher UV reflection may have evolved as an adaptation to discourage bee visitation, but that also enhances flower detectability by birds.

In certain hues, UV reflection enhances detectability because it increases overall brightness (Chittka & Thomson 2001; Kevan *et al*. 2001; Chittka & Raine 2006). Brightness may be particularly important for birds as they possess double cones that are specialized in perceiving light intensity (Hart 2001). In this context, the OUMV model also revealed a higher evolutionary rate of change in UV reflection (σ^2^) in white bee‐flowers than in white bird‐flowers. Therefore, the strength of stabilizing selection was likely stronger for white bird‐flowers (Hansen 1997; Butler & King 2004; Beaulieu *et al*. 2012). This may be due to both sensory exclusion of bees and attraction of birds resulting in the same selective pressure towards enhanced UV reflection in white bird‐flowers. Nevertheless, it is important to note that the role of UV in bird vision has been scarcely investigated in the context of foraging for floral resources (Cuthill *et al*. 2000; Kelber *et al*. 2003; Lunau *et al*. 2011).

Contrary to expectations, we found higher UV reflection optima in yellow bird‐flowers compared to yellow bee‐flowers. In yellow flowers, intra‐floral patterns of UV reflection visually guide bees to floral resources (Lunau 1993; Lunau *et al*. 1996; Papiorek *et al*. 2016; Tunes *et al*. 2021). Such intra‐floral pattern may reduce overall UV reflection when considering the whole flower, as considered in this study, leading to the association between lower UV reflection and yellow bee‐flowers. Nevertheless, yellow bee‐flowers showed higher chromatic contrast in the bee visual system even when compared to red and white bee‐flowers. Thus, intra‐floral UV patterns may still produce a remarkable contrast, despite its lower overall UV reflection. In contrast, yellow bird‐flowers lack intra‐floral UV patterns (Papiorek *et al*. 2016; Camargo *et al*. 2019). Our results suggest that yellow UV‐reflecting bird‐flowers may be more common than expected. Yellow UV‐reflecting is a non‐spectral colour in the bird visual system (i.e., produced by non‐adjacent colour wavebands), which is highly distinguishable to birds (Stoddard *et al*. 2020).

We also found a higher selective strength (α) in bee‐flowers (both in yellow and in other colours). This indicates a rapid shift towards the UV‐reflecting optima and may reflect fewer phenotypic constraints (Hansen 1997; Butler & King 2004; Beaulieu *et al*. 2012), possibly due to enhanced selective pressures in response to bee attraction. Moreover, yellow flowers showed the highest UV reflection optima among bee‐flowers even when compared to red and white flowers. This reinforces the key role of UV reflection in yellow bee‐flowers.

By investigating a wide range of bee‐ and bird‐flowers, we have provided insights into the mechanisms driving the evolution of floral UV reflection. Our results suggest that both the attraction of primary pollinators and the exclusion of less effective floral visitors and antagonists are important processes shaping the evolution of floral UV reflection. We found clear evolutionary signatures of these two processes in red flowers, both in terms of adaptative optima and evolutionary rates of change. However, white and yellow flowers exhibited fewer clear patterns, primarily indicated by the variance in the optima in white bird‐flowers and by higher selective strength in yellow bee‐flowers. Such differences in the evolution of UV reflection have important consequences for plant–pollinator communication by determining the chromatic contrast of flowers to bees and birds. Overall, our findings support that positive and negative interactions have contributed to the evolution of the remarkable diversity of flower colours found in the angiosperms. Despite the limited number of species relative to total angiosperm diversity, our findings offer important initial insights into how floral UV reflection evolves under contrasting pollinator‐driven selective pressures. Future studies should expand taxonomic and ecological coverage to validate and build upon these results.

## AUTHOR CONTRIBUTIONS

LCO, PJB, VLGB, and FJT conceived and designed the study; KL, SG, and LCO collected data; LCO, PJB, and VLGB analysed data; and LCO, PJB, VLGB, KL, SG, PO, and LRFM wrote and revised the manuscript.

## CONFLICT OF INTEREST STATEMENT

The authors declare no conflict of interest.

## Supporting information


**Table S1.** List of 245 plant species selected for macroevolutionary analyses, based on UV reflection data from Klaus Lunau and Sarah Gerten (KL and SG) and Coimbra *et al*. (2020). The table includes botanical classification, attractant structures (S), geographic distribution (N – Neotropical, EN – Extra‐Neotropical, G – Global), pollination system (PS), assigned hue categories, and corresponding UV reflection values (following Camargo *et al*. 2019; Coimbra *et al*. 2020). Attractant structures (S) are classified as follows: P – petal, SP – sepal, B – bract, ST – stamen. Visitor Groups (VG) categorize floral visitors based on their specific behaviour, size, and function during flower visits. Pollination systems (PS) reflect functional pollination strategies based on the main effective pollinator(s) documented in the literature. When conflicting reports were found, we adopted the most frequently cited effective pollinator. Hue categories include 16 distinct combinations, distinguishing UV‐absorbing (UV−) and UV‐reflecting (UV+) types. The dataset spans 180 genera and 71 plant families, representing a broad phylogenetic and ecological range relevant for macroevolutionary inference.
**Table S2.** Evolutionary models with AIC and transition rates values. To perform this reconstruction, we first analyse which of the following evolutionary models best fits the data on “white‐red” flowers: ER (equal rates), ARD (all different rates), and SYM (symmetrical). The best model was selected by comparing Akaike weights (AICc) and ΔAIC. The values of transition rates are: W (white), R (red) and O (Other) and the trace between them indicates the direction of change of state. Highlighted in grey shade is the selected model.
**Table S3.** AICc values of evolutionary models with values of transition rates. To perform this reconstruction, we first analyse which of the following evolutionary models best fits the data on “yellow” flowers: ER (equal rates), ARD (all different rates), and SYM (symmetrical). The best model was selected by comparing Akaike weights (AICc) and ΔAIC. The values of transition rates are: Y (yellow) and O (Other) and the trace between them indicates the direction of change of state. Highlighted in grey shade is the selected model.
**Table S4.** Results of the analysis of variance (ANOVA type II) for generalized linear models (GLM) adjusted to evaluate the effect of pollinator type (bee vs. bird) and hue category (red, white, yellow, and others) on flower chromatic contrast. Two models were tested: one considering the response to chromatic contrast in the bee visual system (Model I) and another considering the response to chromatic contrast in the bird visual system (Model II). Significance codes: ***P < 0.001; **P < 0.01; *P < 0.05; *P* < 0.10.
**Table S5.** Comparisons of estimated marginal means for combinations of flower hue categories (red, white, yellow, other) in the generalized linear model (GLM) for chromatic contrast in the bee visual system. Highlighted in bold is the comparison where the *P*‐values are statistically significant.
**Table S6.** Comparisons of estimated marginal means for combinations of pollinator type (bees vs. birds) and flower hue categories (red, white, yellow, other) in the generalized linear model (GLM) for chromatic contrast in the bird visual system. Highlighted in bold is the comparison where the *P*‐values are statistically significant.
**Table S7.** Comparisons of estimated marginal means for combinations of pollinator system and hue categories (white‐red flowers subset) in the generalized linear mixed model (GLMM) fitted to investigate the interactive effects of pollinator type and hue category on UV reflection optima phenotype values (θ).
**Table S8.** Comparisons of estimated marginal means for combinations of pollinator system and hue categories (yellow flowers subset) in the generalized linear mixed model (GLMM) fitted to investigate the interactive effects of pollinator type and hue category on UV reflection optima phenotype values (θ). Highlighted in bold is the comparison where the *P*‐values are statistically significant.

**Fig. S1.** Smoothed average reflectance spectra of flower colours from the species analysed. The colours of the flowers were categorized based on their reflection in the UV (300‐400 nm), blue (400‐500 nm), green (500‐600 nm) and red (600–700) wavelengths. Established thresholds for mean spectral reflection were 0.1 (10%) for UV, 0.3 (30%) for blue, 0.4 (40%) for green, 0.5 (50%) to distinguish green flowers from blue and red ones, and 0.6 (60%) for red (adapted from Camargo *et al*. 2019 and Coimbra *et al*. 2020). The red line highlights the UV threshold (0.1) to differentiate reflectance patterns of UV versus non‐UV flowers. Reflectance patterns average of non‐UV (UV−) colours are represented on the left, and those of UV (UV+) colours are represented on the right.
**Fig. S2.** Reflectance spectra of red, yellow, and white flowers pollinated by bees (left) and birds (right) from the analysed species. The red line indicates the UV threshold (0.1), used to distinguish UV‐reflective (UV+) from non‐UV‐reflective (UV−) flowers.
**Fig. S3.** Estimated Marginal Means of chromatic contrast based on the bee visual system, considering (A) pollinator type (bee vs. bird) and (B) flower hue categories (red, white, yellow, and others). The points represent the mean estimated marginal means for each pollinator type (A) and hue category (B), while the error bars indicate the 95% confidence intervals. The figure highlights potential differences in chromatic contrast across hue categories and as perceived by different types of pollinators Full statistical results are provided in Tables S7 and S8.
**Fig. S4.** Estimated Marginal Means of chromatic contrast based on the bird visual system as a function of pollinator type (bee vs. bird) and flower hue category (white, red, yellow, other). The points represent the mean estimated marginal means for each combination of pollinator type and hue category. The graph highlights how chromatic contrast varies across different hue categories depending on the pollinator type. Full statistical results are provided in Tables S7 and S8.
**Fig. S5.** Residual Diagnostics for the GLMM investigating the interactive effects of pollinator type (bee vs bird) and hue category on optima phenotype (θ) of white‐red flowers. Additionally, the results of Kolmogorov–Smirnov (KS), dispersion and outlier statistical tests are provided.
**Fig. S6.** Residual Diagnostics for the GLMM investigating the interactive effects of pollinator type (bee vs bird) and hue category on optima phenotype (θ) of yellow flowers. Additionally, the results of Kolmogorov–Smirnov (KS), dispersion and outlier statistical tests are provided.

## Data Availability

All datasets supporting the results of this study are included within the Supporting Information.
