# Supplementary material for: Evolution of UV reflection in bee‐ and bird‐pollinated flowers
Source: Plant Biol (Stuttg). 2025 Nov 14;28(1):201–14. doi: 10.1111/plb.70138 (PMC12710832; doi:10.1111/plb.70138)
Supplement: Supplementary file 1 — Table S1. List of 245 plant species selected for macroevolutionary analyses, based on UV reflection data from Klaus Lunau and Sarah Gerten (KL and SG) and Coimbra et al. (2020). The table includes botanical classification, attractant structures (S), geographic distribution (N – Neotropical, EN – Extra‐Neotropical, G – Global), pollination system (PS), assigned hue categories, and corresponding UV reflection values (following Camargo et al. 2019; Coimbra et al. 2020). Attractant structures (S) are classified as follows: P – petal, SP – sepal, B – bract, ST – stamen. Visitor Groups (VG) categorize floral visitors based on their specific behaviour, size, and function during flower visits. Pollination systems (PS) reflect functional pollination strategies based on the main effective pollinator(s) documented in the literature. When conflicting reports were found, we adopted the most frequently cited effective pollinator. Hue categories include 16 distinct combinations, distinguishing UV‐absorbing (UV−) and UV‐reflecting (UV+) types. The dataset spans 180 genera and 71 plant families, representing a broad phylogenetic and ecological range relevant for macroevolutionary inference. Table S2. Evolutionary models with AIC and transition rates values. To perform this reconstruction, we first analyse which of the following evolutionary models best fits the data on “white‐red” flowers: ER (equal rates), ARD (all different rates), and SYM (symmetrical). The best model was selected by comparing Akaike weights (AICc) and ΔAIC. The values of transition rates are: W (white), R (red) and O (Other) and the trace between them indicates the direction of change of state. Highlighted in grey shade is the selected model. Table S3. AICc values of evolutionary models with values of transition rates. To perform this reconstruction, we first analyse which of the following evolutionary models best fits the data on “yellow” flowers: ER (equal rates), ARD (all different rates), and SYM (symmetrica [file PLB-28-201-s001.docx]

## Supplementar tables

**Table S1** List of 245 plant species selected for macroevolutionary analyses, based on UV reflection data from Klaus Lunau and Sarah Gerten (KL and SG) and Coimbra et al. (2020). The table includes botanical classification, attractant structures (S), geographic distribution (N – Neotropical, EN – Extra-Neotropical, G – Global), pollination system (PS), assigned hue categories, and corresponding UV reflection values (following Camargo et al., 2019; Coimbra et al., 2020). Attractant structures (S) are classified as follows:P – petal, SP – sepal, B – bract, ST – stamen. Visitor Groups (VG) categorize floral visitors based on their specific behavior, size, and function during flower visits. Pollination systems (PS) reflect functional pollination strategies based on the main effective pollinator(s) documented in the literature. When conflicting reports were found, we adopted the most frequently cited effective pollinator. Hue categories include 16 distinct combinations, distinguishing UV-absorbing (UV−) and UV-reflecting (UV+) types. The dataset spans 180 genera and 71 plant families, representing a broad phylogenetic and ecological range relevant for macroevolutionary inference.

| **Species** | **Genus** | **Family** | **S** | **Area** | **VG** | **PS** | **Hue** | **UV reflection** | **RI** | **Database** |
| --- | --- | --- | --- | --- | --- | --- | --- | --- | --- | --- |
| *Zephyranthes candida* (Lindl.) Herb. | Zephyranthes | Amaryllidaceae | P | EN | bee | bee | UV-White | 0.024 | 16 | KL and SG dataset |
| *Allamanda blanchetii* A.DC. | Allamanda | Apocynaceae | P | G | bee | bee | UV-Pink | 0.051 | 19 | KL and SG dataset |
| *Allamanda schottii* Pohl | Allamanda | Apocynaceae | P | G | bee | bee | UV-Yellow | 0.038 | 21 | KL and SG dataset |
| *Gomphocarpus physocarpus* E.Mey. | Gomphocarpus | Apocynaceae | P | G | wasp | bee | UV+White | 0.055 | 149 | KL and SG dataset |
| *Pachypodium bispinosum* (L.f.) A.DC. | Pachypodium | Apocynaceae | P | EN | bee | bee | UV+White | 0.063 | 17 | KL and SG dataset |
| *Spathiphyllum cannifolium* (Dryand. ex Sims) Schott | Spathiphyllum | Araceae | B | N | bee | bee | UV+White | 0.736 | 278 | KL and SG dataset |
| *Leopoldia longipes* (Boiss.) Losinsk. | Leopoldia | Asparagaceae | P | EN | bee | bee | UV-Blue | 0.048 | 1 | Coimbra et al. 2020 |
| *Aloe africana* Mill. | Aloe | Asphodelaceae | P | EN | bird | bird | UV-Yellow | 0.044 | 22 | KL and SG dataset |
| *Aloe bakeri* Scott-Elliot | Aloe | Asphodelaceae | P | EN | bird | bird | UV+Yellow | 0.138 | 16 | KL and SG dataset |
| *Aloe bellatula* Reynolds | Aloe | Asphodelaceae | P | EN | bird | bird | UV+Red | 0.071 | 16 | KL and SG dataset |
| *Aloe ciliaris* Haw. | Aloe | Asphodelaceae | P | EN | bird | bird | UV-Red | 0.056 | 16 | KL and SG dataset |
| *Aloe descoingsii* Reynolds | Aloe | Asphodelaceae | P | EN | bird | bird | UV+Red | 0.074 | 16 | KL and SG dataset |
| *Aloe plicatilis* (L.) Mill. | Aloe | Asphodelaceae | P | EN | bird | bird | UV+Red | 0.047 | 16 | KL and SG dataset |
| *Aloe viguieri* H.Perrier | Aloe | Asphodelaceae | P | EN | bird | bird | UV+Red | 0.153 | 16 | KL and SG dataset |
| *Aloe vogtsii* Reynolds | Aloe | Asphodelaceae | P | EN | bird | bird | UV-Yellow | 0.031 | 23 | KL and SG dataset |
| *Asphodeline liburnica* (Scop.) Rchb. | Asphodeline | Asphodelaceae | P | EN | bee | bee | UV+Yellow | 0.088 | 34 | KL and SG dataset |
| *Asphodelus aestivus* Brot. | Asphodelus | Asphodelaceae | P | EN | bee | bee | UV+White | 0.089 | 34 | KL and SG dataset |
| *Eremurus stenophyllus* (Boiss. & Buhse) Baker | Eremurus | Asphodelaceae | P | EN | bee | bee | UV+Yellow | 0.061 | 16 | KL and SG dataset |
| *Gasteria pulchra* (Aiton) Haw. | Gasteria | Asphodelaceae | P | EN | bird | bird | UV-Red | 0.007 | 170 | Coimbra et al. 2020 |
| *Kniphofia uvaria* (L.) Oken | Kniphofia | Asphodelaceae | P | EN | bird | bird | UV-Red | 0.043 | 274 | KL and SG dataset |
| *Baccharis trimera* (Less.) DC. | Baccharis | Asteraceae | P | N | bee | bee | UV-Yellow | 0.015 | 12 | KL and SG dataset |
| *Bidens ferulifolia* (Jacq.) Sweet | Bidens | Asteraceae | P | EN | bee | bee | UV+Yellow | 0.089 | 43 | KL and SG dataset |
| *Centaurea aegyptiaca* L. | Centaurea | Asteraceae | P | EN | bee | bee | UV+White | 0.05 | 1 | Coimbra et al. 2020 |
| *Centaurea ammocyanus* Boiss. | Centaurea | Asteraceae | P | EN | bee | bee | UV+Pink | 0.062 | 1 | Coimbra et al. 2020 |
| *Centaurea pallescens* Delile | Centaurea | Asteraceae | P | EN | bee | bee | UV-White | 0.005 | 1 | Coimbra et al. 2020 |
| *Cirsium oleraceum* (L.) Scop. | Cirsium | Asteraceae | P | EN | bee | bee | UV-White | 0.017 | 1 | Coimbra et al. 2020 |
| *Coreopsis grandiflora* Hogg ex Sweet | Coreopsis | Asteraceae | P | EN | bee | bee | UV-Yellow | 0.045 | 89 | KL and SG dataset |
| *Emilia coccinea* (Sims) G.Don | Emilia | Asteraceae | P | EN | bee | bee | UV+White | 0.248 | 186 | KL and SG dataset |
| *Erigeron canadensis* L. | Erigeron | Asteraceae | P | EN | bee | bee | UV-White | 0.033 | 1 | Coimbra et al. 2020 |
| *Mutisia acuminata* Ruiz & Pav. | Mutisia | Asteraceae | P | N | bird | bird | UV+Red | 0.088 | 203 | KL and SG dataset |
| *Mutisia coccinea* A.St.-Hil. | Mutisia | Asteraceae | P | N | bird | bird | UV-Red | 0.042 | 244 | KL and SG dataset |
| *Senecio inaequidens* DC. | Senecio | Asteraceae | P | EN | bee | bee | UV-Yellow | 0.047 | 16 | KL and SG dataset |
| *Senecio inaequidens* DC*.* | Senecio | Asteraceae | P | EN | bee | bee | UV+Yellow | 0.167 | 16 | KL and SG dataset |
| *Stifftia chrysantha* J.C.Mikan | Stifftia | Asteraceae | P | N | bird | bird | UV-Yellow | 0.015 | 207 | KL and SG dataset |
| *Impatiens niamniamensis* Gilg | Impatiens | Balsamiaceae | P | EN | bird | bird | UV-Red | 0.031 | 52 | KL and SG dataset |
| *Impatiens noli-tangere* L. | Impatiens | Balsamiaceae | P | EN | bee | bee | UV+Yellow | 0.236 | 229 | KL and SG dataset |
| *Impatiens scabrida* DC. | Impatiens | Balsamiaceae | P | EN | bee | bee | UV-Yellow | 0.051 | 230 | KL and SG dataset |
| *Begonia coccinea* Hook. | Begonia | Begoniaceae | P | G | bee | bee | UV+White | 0.12 | 40 | KL and SG dataset |
| *Berberis darwinii* Hook. | Berberis | Berberidaceae | P | EN | bee | bee | UV+Yellow | 0.142 | 41 | KL and SG dataset |
| *Epimedium versicolor* E.Morren | Epimedium | Berberidaceae | P | EN | bee | bee | UV+Yellow | 0.08 | 260 | KL and SG dataset |
| *Corylus avellana* L. | Corylus | Bertulaceae | P | EN | bee | bee | UV-Yellow | 0.011 | 1 | Coimbra et al. 2020 |
| *Campsis grandiflora* (Thunb.) K.Schum. | Campsis | Bignoniaceae | P | EN | bee | bee | UV+Red | 0.086 | 59 | KL and SG dataset |
| *Campsis radicans* (L.) Seem. | Campsis | Bignoniaceae | P | EN | bird | bird | UV-Red | 0.082 | 17 | KL and SG dataset |
| *Clytostoma callistegioides* (Cham.) Baill. | Clytostoma | Bignoniaceae | P | G | bee | bee | UV-Pink | 0.053 | 78 | KL and SG dataset |
| *Handroanthus heptaphyllus* (Vell.) Mattos | Handroanthus | Bignoniaceae | P | G | bee | bee | UV-Pink | 0.02 | 78 | KL and SG dataset |
| *Incarvillea delavayi* Bureau & Franch. | Incarvillea | Bignoniaceae | P | EN | bee | bee | UV+Pink | 0.08 | 137 | KL and SG dataset |
| *Pandorea jasminoides* (Lindl.) K.Schum*.* | Pandorea | Bignoniaceae | P | EN | bee | bee | UV-Pink | 0.101 | 222 | KL and SG dataset |
| *Spathodea campanulata* P.Beauv. | Spathodea | Bignoniaceae | P | EN | bird | bird | UV-Red | 0.084 | 265 | KL and SG dataset |
| *Zeyheria montana* Mart. | Zeyheria | Bignoniaceae | P | N | bird | bird | UV+Yellow | 0.287 | 121 | KL and SG dataset |
| *Cochlospermum vitifolium* (Willd.) Spreng. | Cochlospermum | Bixaceae | P | N | bee | bee | UV+Yellow | 0.128 | 80 | KL and SG dataset |
| *Alkanna strigosa* Boiss. & Hohen. | Alkanna | Boraginaceae | P | G | bee | bee | UV+Blue | 0.109 | 1 | Coimbra et al. 2020 |
| *Anchusa strigosa* Banks & Sol. | Anchusa | Boraginaceae | P | EN | bee | bee | UV+Blue | 0.112 | 1 | Coimbra et al. 2020 |
| *Echium angustifolium* Mill. | Echium | Boraginaceae | P | EN | bee | bee | UV-Pink | 0.131 | 1 | Coimbra et al. 2020 |
| *Echium candicans* L.f. | Echium | Boraginaceae | P | EN | bee | bee | UV-Blue | 0.024 | 271 | KL and SG dataset |
| *Echium hierrense* Webb ex Bolle | Echium | Boraginaceae | P | EN | bee | bee | UV+Pink | 0.071 | 140 | KL and SG dataset |
| *Echium vulgare* L. | Echium | Boraginaceae | P | EN | bee | bee | UV+Blue | 0.151 | 47 | KL and SG dataset |
| *Symphytum brachycalyx* Boiss. | Symphytum | Boraginaceae | P | EN | bee | bee | UV-White | 0.008 | 1 | Coimbra et al. 2020 |
| *Hesperis pendula* DC. | Hesperis | Brassicaceae | P | EN | bee | bee | UV-Green | 0.016 | 1 | Coimbra et al. 2020 |
| *Moricandia nitens* (Viv.) E.A.Durand & Barratte | Moricandia | Brassicaceae | P | EN | bee | bee | UV+Pink | 0.147 | 1 | Coimbra et al. 2020 |
| *Zilla spinosa* (L.) Prantl | Zilla | Brassicaceae | P | EN | bee | bee | UV-Pink | 0.044 | 1 | Coimbra et al. 2020 |
| *Aechmea aquilega* (Salisb.) Griseb. | Aechmea | Bromeliaceae | P | G | bird | bird | UV-Red | 0.008 | 3 | Coimbra et al. 2020 |
| *Aechmea blanchetiana* (Baker) L.B.Sm. | Aechmea | Bromeliaceae | B | N | bird | bird | UV-Red | 0.013 | 9 | KL and SG dataset |
| *Aechmea fulgens* Brongn. | Aechmea | Bromeliaceae | P | N | bird | bird | UV+Pink | 0.091 | 13,27 | KL and SG dataset |
| *Aechmea pectinata* Baker | Aechmea | Bromeliaceae | P | N | bird | bird | UV+White | 0.058 | 15 | KL and SG dataset |
| *Aechmea recurvata* (Klotzsch) L.B.Sm. | Aechmea | Bromeliaceae | P | N | bird | bird | UV+Pink | 0.258 | 16 | KL and SG dataset |
| *Ananas bracteatus* (Lindl.) Schult. & Schult.f. | Ananas | Bromeliaceae | B | N | bird | bird | UV-Red | 0.038 | 27 | KL and SG dataset |
| *Billbergia amoena* (Lodd.) Lindl. | Billbergia | Bromeliaceae | P | N | bird | bird | UV+Green | 0.166 | 44,27 | KL and SG dataset |
| *Billbergia nutans* H.Wendl. ex Regel | Billbergia | Bromeliaceae | B | G | bird | bird | UV-Red | 0.046 | 16 | KL and SG dataset |
| *Billbergia pyramidalis* (Sims) Lindl. | Billbergia | Bromeliaceae | B | G | bird | bird | UV-Red | 0.004 | 45,27 | KL and SG dataset |
| *Billbergia viridiflora* H.Wendl. | Billbergia | Bromeliaceae | B | N | bird | bird | UV+Red | 0.175 | 16 | KL and SG dataset |
| *Fosterella penduliflora* (C.H.Wright) L.B.Sm. | Fosterella | Bromeliaceae | P | N | bee | bee | UV-White | 0.043 | 16 | KL and SG dataset |
| *Neoregelia cruenta* (Graham) L.B.Sm. | Neoregelia | Bromeliaceae | P | N | bird | bird | UV-Blue | 0.034 | 146 | Coimbra et al. 2020 |
| *Pitcairnia heterophylla* (Lindl.) Beer | Pitcairnia | Bromeliaceae | P | N | bird | bird | UV+Red | 0.122 | 16 | KL and SG dataset |
| *Puya santosii* Cuatrec. | Puya | Bromeliaceae | P | EN | bird | bird | UV+Cyan | 0.143 | 16 | KL and SG dataset |
| *Quesnelia arvensis* (Vell.) Mez | Quesnelia | Bromeliaceae | B | N | bird | bird | UV-Pink | 0.017 | 45 | Coimbra et al. 2020 |
| *Quesnelia liboniana* (De Jonghe) Mez | Quesnelia | Bromeliaceae | P | N | bird | bird | UV+Blue | 0.11 | 202 | Coimbra et al. 2020 |
| *Tillandsia aeranthos* (Loisel.) L.B.Sm. | Tillandsia | Bromeliaceae | P | G | bird | bird | UV-Blue | 0.124 | 253,27 | KL and SG dataset |
| *Tillandsia ionantha* Planch. | Tillandsia | Bromeliaceae | B | G | bird | bird | UV+Blue | 0.177 | 154 | KL and SG dataset |
| *Vriesea neoglutinosa* Mez | Vriesea | Bromeliaceae | B | N | bird | bird | UV-Red | 0 | 146,27 | KL and SG dataset |
| *Mammillaria sheldonii* (Britton & Rose) Boed. | Mammillaria | Cactaceae | P | N | bee | bee | UV+Pink | 0.139 | 2 | KL and SG dataset |
| *Opuntia fragilis* (Nutt.) Haw. | Opuntia | Cactaceae | P | EN | bird | bird | UV+Pink | 0.191 | 197 | KL and SG dataset |
| *Rhipsalis baccifera* (J.S.Muell.) Stearn | Rhipsalis | Cactaceae | P | G | bird | bird | UV+White | 0.06 | 27,122 | KL and SG dataset |
| *Rhipsalis elliptica* G.Lindb. ex K.Schum. | Rhipsalis | Cactaceae | P | N | bee | bee | UV+White | 0.047 | 122 | KL and SG dataset |
| *Tacinga palmadora* (Britton & Rose) N.P.Taylor & Stuppy | Tacinga | Cactaceae | P | N | bird | bird | UV-Red | 0.01 | 191 | KL and SG dataset |
| *Campanula cochleariifolia* Lam. | Campanula | Campanulaceae | P | EN | bee | bee | UV+Blue | 0.249 | 57 | KL and SG dataset |
| *Campanula poscharskyana* Degen | Campanula | Campanulaceae | P | EN | bee | bee | UV+Blue | 0.093 | 58,62 | KL and SG dataset |
| *Canarina canariensis* (L.) Vatke | Canarina | Campanulaceae | P | EN | bird | bird | UV+Red | 0.136 | 60 | KL and SG dataset |
| *Centropogon cornutus* (L.) Druce | Centropogon | Campanulaceae | P | G | bird | bird | UV-Red | 0.057 | 45 | KL and SG dataset |
| *Centropogon valerii* Standl. | Centropogon | Campanulaceae | P | N | bird | bird | UV-Red | 0.052 | 66 | KL and SG dataset |
| *Lobelia anceps* L.f. | Lobelia | Campanulaceae | P | EN | bee | bee | UV-Blue | 0.003 | 17 | Coimbra et al. 2020 |
| *Lobelia siphilitica* L. | Lobelia | Campanulaceae | P | EN | bee | bee | UV+Blue | 0.074 | 172 | KL and SG dataset |
| *Lobelia tupa* L. | Lobelia | Campanulaceae | P | N | bird | bird | UV+Red | 0.135 | 204,206 | KL and SG dataset |
| *Leycesteria formosa* Wall. | Leycesteria | Caprifoliaceae | P | EN | bee | bee | UV-White | 0.035 | 17 | KL and SG dataset |
| *Lonicera fragrantissima* Lindl. & J. Paxton | Lonicera | Caprifoliaceae | P | EN | bee | bee | UV+White | 0.053 | 256 | KL and SG dataset |
| *Moehringia trinervia* (L.) Clairv. | Moehringia | Caryophyllaceae | P | EN | bee | bee | UV-White | 0.021 | 1 | Coimbra et al. 2020 |
| *Silene aegyptiaca* (L.) L.f. | Silene | Caryophyllaceae | P | EN | bee | bee | UV+Pink | 0.071 | 1 | Coimbra et al. 2020 |
| *Cistus creticus* (Viv.) Greuter & Burdet | Cistus | Cistaceae | P | EN | bee | bee | UV-Pink | 0.009 | 73 | KL and SG dataset |
| *Clusia lanceolata* Cambess. | Clusia | Clusiaceae | P | N | bee | bee | UV+White | 0.252 | 77 | KL and SG dataset |
| *Desfontainia spinosa* Ruiz & Pav. | Desfontainia | Columelliaceae | P | N | bird | bird | UV-Red | 0.039 | 107 | KL and SG dataset |
| *Dichorisandra thyrsiflora* J.C.Mikan | Dichorisandra | Commelinaceae | P | EN | bee | bee | UV+Blue | 0.116 | 255 | KL and SG dataset |
| *Costus afer* Ker Gawl. | Costus | Costaceae | P | EN | bee | bee | UV+White | 0.384 | 42 | KL and SG dataset |
| *Costus erythrophyllus* Loes. | Costus | Costaceae | P | N | bee | bee | UV+White | 0.197 | 92 | KL and SG dataset |
| *Costus malortieanus* H.Wendl. | Costus | Costaceae | P | G | bee | bee | UV+Yellow | 0.092 | 94 | KL and SG dataset |
| *Costus pulverulentus* C.Presl | Costus | Costaceae | P | N | bird | bird | UV+Red | 0.14 | 94 | KL and SG dataset |
| *Costus spicatus* (Jacq.) Sw. | Costus | Costaceae | P | N | bird | bird | UV-Pink | 0.025 | 94 | KL and SG dataset |
| *Hellenia speciosa* (J.Koenig) S.R.Dutta | Hellenia | Costaceae | P | G | bee | bee | UV+White | 0.124 | 242 | KL and SG dataset |
| *Crinodendron patagua* Molina | Crinodendron | Elaeocarpaceae | P | N | bee | bee | UV-White | 0.033 | 95 | KL and SG dataset |
| *Agapetes hosseana* Diels | Agapetes | Ericaceae | P | EN | bird | bird | UV-Red | 0.167 | 17 | KL and SG dataset |
| *Agapetes serpens* (Wight) Sleumer | Agapetes | Ericaceae | P | EN | bird | bird | UV+Red | 0.107 | 17 | KL and SG dataset |
| *Arctostaphylos uva-ursi* (L.) Spreng. | Arctostaphylos | Ericaceae | P | EN | bee | bee | UV-White | 0.033 | 1 | Coimbra et al. 2020 |
| *Erica caffra* L. | Erica | Ericaceae | P | EN | bee | bee | UV+White | 0.061 | 17 | KL and SG dataset |
| *Erica versicolor* Andrews | Erica | Ericaceae | P | EN | bird | bird | UV-Red | 0.006 | 236 | KL and SG dataset |
| *Pieris japonica* (Thunb.) D. Don ex G. Don | Pieris | Ericaceae | P | EN | bee | bee | UV-White | 0.031 | 261 | KL and SG dataset |
| *Vaccinium vitis-idaea* L. | Vaccinium | Ericaceae | P | EN | bee | bee | UV-White | 0.017 | 1 | Coimbra et al. 2020 |
| *Dalechampia spathulata* (Scheidw.) Baill. | Dalechampia | Euphorbiaceae | L | N | bee | bee | UV+Pink | 0.069 | 104 | KL and SG dataset |
| *Euphorbia pulcherrima* Willd. ex Klotzsch | Euphorbia | Euphorbiaceae | B | N | bird | bird | UV-Red | 0.101 | 207 | KL and SG dataset |
| *Jatropha podagrica* Hook. | Jatropha | Euphorbiaceae | P | EN | bird | bird | UV-Red | 0.041 | 2 | KL and SG dataset |
| *Amherstia nobilis* Wall. | Amherstia | Fabaceae | P | EN | bird | bird | UV-Red | 0.058 | 47 | KL and SG dataset |
| *Anagyris foetida* L. | Anagyris | Fabaceae | P | EN | bee | bee | UV-Yellow | 0.056 | 1 | Coimbra et al. 2020 |
| *Astragalus amalecitanus* Boiss. | Astragalus | Fabaceae | P | EN | bee | bee | UV-White | 0.024 | 1 | Coimbra et al. 2020 |
| *Calicotome villosa* (Poir.) Link | Calicotome | Fabaceae | P | EN | bee | bee | UV+Yellow | 0.06 | 1 | Coimbra et al. 2020 |
| *Cercis siliquastrum* L. | Cercis | Fabaceae | P | EN | bee | bee | UV-Pink | 0.013 | 1 | Coimbra et al. 2020 |
| *Collaea argentina* Griseb. | Collaea | Fabaceae | P | N | bee | bee | UV+Pink | 0.073 | 85 | KL and SG dataset |
| *Crotalaria agatiflora* Schweinf. | Crotalaria | Fabaceae | P | EN | bird | bird | UV+Green | 0.1 | 97 | KL and SG dataset |
| *Dahlstedtia pinnata* (Benth.) Malme | Dahlstedtia | Fabaceae | P | N | bird | bird | UV+Pink | 0.071 | 45 | Coimbra et al. 2020 |
| *Erythrina falcata* Benth. | Erythrina | Fabaceae | P | N | bird | bird | UV-Red | 0.023 | 143 | KL and SG dataset |
| *Erythrina fusca* Lour. | Erythrina | Fabaceae | P | G | bird | bird | UV+White | 0.054 | 209 | KL and SG dataset |
| *Erythrina humeana* Spreng. | Erythrina | Fabaceae | P | G | bird | bird | UV-Red | 0.087 | 17 | KL and SG dataset |
| *Erythrina speciosa* Andrews | Erythrina | Fabaceae | P | N | bird | bird | UV+Red | 0.094 | 45 | Coimbra et al. 2020 |
| *Lessertia frutescens* (L.) Goldblatt & J.C. Manning | Lessertia | Fabaceae | P | EN | bird | bird | UV+Red | 0.102 | 249 | KL and SG dataset |
| *Lotus berthelotii* Masf. | Lotus | Fabaceae | P | EN | bird | bird | UV-Red | 0.064 | 60 | KL and SG dataset |
| *Macroptilium atropurpureum* (DC.) Urb. | Macroptilium | Fabaceae | P | G | bee | bee | UV+Black | 0.256 | 272 | KL and SG dataset |
| *Senna artemisioides* Isely | Senna | Fabaceae | P | EN | bee | bee | UV-Yellow | 0.054 | 200 | KL and SG dataset |
| *Senna bicapsularis* (L.) Roxb. | Senna | Fabaceae | P | G | bee | bee | UV+Yellow | 0.187 | 200 | KL and SG dataset |
| *Swartzia oblata* Cowan | Swartzia | Fabaceae | P | EN | bee | bee | UV-White | 0.033 | 228 | KL and SG dataset |
| *Swartzia simplex* (Sw.) Spreng. | Swartzia | Fabaceae | P | N | bee | bee | UV+Yellow | 0.219 | 228 | Coimbra et al. 2020 |
| *Vicia faba* L. | Vicia | Fabaceae | P | EN | bee | bee | UV+White | 0.064 | 150 | KL and SG dataset |
| *Gentiana asclepiadea* L. | Gentiana | Gentianaceae | P | EN | bee | bee | UV+Blue | 0.171 | 218 | KL and SG dataset |
| *Pelargonium crithmifolium* Sm. | Pelargonium | Geraniaceae | P | EN | bee | bee | UV+White | 0.092 | 259 | KL and SG dataset |
| *Codonanthe gracilis* (Mart.) Hanst. | Codonanthe | Gesneriaceae | P | N | bee | bee | UV+White | 0.059 | 17 | KL and SG dataset |
| *Columnea magnifica* Klotzsch ex Oerst. | Columnea | Gesneriaceae | P | N | bird | bird | UV+Red | 0.104 | 86 | KL and SG dataset |
| *Columnea microcalyx* Hanst. | Columnea | Gesneriaceae | P | N | bird | bird | UV-Red | 0.052 | 86 | KL and SG dataset |
| *Gesneria ventricosa* Sw. | Gesneria | Gesneriaceae | P | N | bird | bird | UV-Red | 0.045 | 17 | KL and SG dataset |
| *Kohleria spicata* (Kunth) Oerst. | Kohleria | Gesneriaceae | P | N | bird | bird | UV+Red | 0.124 | 201 | KL and SG dataset |
| *Sinningia aggregata* (Ker Gawl.) Wiehler | Sinningia | Gesneriaceae | P | N | bird | bird | UV+Red | 0.074 | 223 | KL and SG dataset |
| *Sinningia eumorpha* H.E. Moore | Sinningia | Gesneriaceae | P | N | bee | bee | UV+White | 0.045 | 223 | KL and SG dataset |
| *Sinningia macropoda* (Sprague) H.E. Moore | Sinningia | Gesneriaceae | P | N | bird | bird | UV-Red | 0.077 | 223 | KL and SG dataset |
| *Sinningia sellovii* (Mart.) Wiehler | Sinningia | Gesneriaceae | P | N | bird | bird | UV-Red | 0.068 | 223 | KL and SG dataset |
| *Anigozanthos flavidus* DC. | Anigozanthos | Haemodoraceae | P | EN | bird | bird | UV-Cyan | 0.029 | 2 | KL and SG dataset |
| *Heliconia angusta* Vell. | Heliconia | Heliconiaceae | B | G | bird | bird | UV+Red | 0.099 | 45 | KL and SG dataset |
| *Heliconia bihai* (L.) L. | Heliconia | Heliconiaceae | B | N | bird | bird | UV+Red | 0.773 | 158 | KL and SG dataset |
| *Heliconia farinosa* Raddi | Heliconia | Heliconiaceae | B | N | bird | bird | UV-Red | 0.052 | 258 | KL and SG dataset |
| *Heliconia metallica* Planch. & Linden ex Hook. | Heliconia | Heliconiaceae | P | G | bird | bird | UV-Red | 0.074 | 246 | Coimbra et al. 2020 |
| *Heliconia rostrata* Ruiz & Pav. | Heliconia | Heliconiaceae | P | G | bird | bird | UV+Red | 0.068 | 45 | KL and SG dataset |
| *Philadelphus coronarius* L. | Philadelphus | Hydrangeaceae | P | EN | bee | bee | UV+White | 0.042 | 231 | KL and SG dataset |
| *Chasmanthe aethiopica* (L.) N.E.Br. | Chasmanthe | Iridaceae | P | EN | bird | bird | UV+Red | 0.07 | 70 | KL and SG dataset |
| *Iris domestica* (L.) Goldblatt & Mabb. | Iris | Iridaceae | P | EN | bee | bee | UV-Red | 0.297 | 190 | KL and SG dataset |
| *Phlomoides tuberosa* (L.) Moench | Phlomoides | Lamiaceae | P | EN | bee | bee | UV+White | 0.07 | 215 | KL and SG dataset |
| *Salvia cacaliifolia* Benth. | Salvia | Lamiaceae | P | N | bird | bird | UV+Blue | 0.085 | 276 | KL and SG dataset |
| *Salvia canariensis* L. | Salvia | Lamiaceae | P | EN | bee | bee | UV+Pink | 0.056 | 277 | KL and SG dataset |
| *Salvia patens* Cav. | Salvia | Lamiaceae | P | G | bird | bird | UV+Blue | 0.113 | 275 | KL and SG dataset |
| *Salvia splendens* Sellow ex Schult. | Salvia | Lamiaceae | P | EN | bird | bird | UV+Red | 0.21 | 255 | KL and SG dataset |
| *Scutellaria costaricana* H.Wendl. | Scutellaria | Lamiaceae | P | N | bird | bird | UV-Red | 0.048 | 17 | KL and SG dataset |
| *Tripora divaricata* (Maxim.) P.D.Cantino | Tripora | Lamiaceae | P | EN | bee | bee | UV+Blue | 0.136 | 263 | KL and SG dataset |
| *Pinguicula alpina* L. | Pinguicula | Lentibulariaceae | P | EN | bee | bee | UV-White | 0.01 | 1 | Coimbra et al. 2020 |
| *Erythronium oregonum* Applegate | Erythronium | Liliaceae | P | EN | bee | bee | UV+White | 0.049 | 217 | KL and SG dataset |
| *Linum grandiflorum* Desf. | Linum | Linaceae | P | EN | bee | bee | UV+Red | 0.293 | 2 | KL and SG dataset |
| *Phragmanthera usuiensis* (Oliv.) M.G.Gilbert | Phragmanthera | Loranthaceae | P | EN | bird | bird | UV+Red | 0.101 | 183 | KL and SG dataset |
| *Byrsonima verbascifolia* (L.) DC. | Byrsonima | Malpighiaceae | P | N | bee | bee | UV+Yellow | 0.636 | 51 | KL and SG dataset |
| *Alcea rosea* L. | Alcea | Malvaceae | P | EN | bee | bee | UV+Pink | 0.065 | 7 | KL and SG dataset |
| *Apeiba tibourbou* Aubl. | Apeiba | Malvaceae | P | N | bee | bee | UV+Yellow | 0.26 | 29 | KL and SG dataset |
| *Hibiscus schizopetalus* (Dyer) Hook.f. | Hibiscus | Malvaceae | P | G | bird | bird | UV+Red | 0.103 | 51 | KL and SG dataset |
| *Malva alcea* L*.* | Malva | Malvaceae | P | EN | bee | bee | UV+Pink | 0.115 | 181 | KL and SG dataset |
| *Napaea dioica* L. | Napaea | Malvaceae | P | EN | bee | bee | UV+White | 0.037 | 169 | KL and SG dataset |
| *Pavonia multiflora* A. St.-Hil. | Pavonia | Malvaceae | SP | N | bird | bird | UV-Red | 0.084 | 17 | KL and SG dataset |
| *Clidemia hirta* (L.) D. Don | Clidemia | Melastomataceae | P | EN | bee | bee | UV-White | 0.033 | 10 | KL and SG dataset |
| *Heterocentron elegans* (Schltdl.) Kuntze | Heterocentron | Melastomataceae | P | N | bee | bee | UV+Pink | 0.21 | 238 | KL and SG dataset |
| *Medinilla magnifica* Lindl. | Medinilla | Melastomataceae | P | EN | bee | bee | UV+Pink | 0.118 | 264 | KL and SG dataset |
| *Melastoma malabathricum* L. | Melastoma | Melastomataceae | P | EN | bee | bee | UV-White | 0.011 | 195 | KL and SG dataset |
| *Ensete lasiocarpum* (Franch.) Cheesman | Musella | Musaceae | B | EN | bee | bee | UV+Yellow | 0.109 | 189 | KL and SG dataset |
| *Callistemon viminalis* (Sol. ex Gaertn.) G.Don | Callistemon | Myrtaceae | ST | EN | bird | bird | UV+Red | 0.115 | 2 | KL and SG dataset |
| *Melaleuca elliptica* Labill. | Melaleuca | Myrtaceae | ST | EN | bird | bird | UV-Red | 0.057 | 2 | KL and SG dataset |
| *Melaleuca hypericifolia* Sm. | Melaleuca | Myrtaceae | P | EN | bird | bird | UV+Red | 0.105 | 226 | KL and SG dataset |
| *Melaleuca quadrifida* (R.Br.) Craven & R.D.Edwards | Melaleuca | Myrtaceae | ST | EN | bird | bird | UV+Red | 0.132 | 129 | KL and SG dataset |
| *Fuchsia boliviana* CarriÃ¨re | Fuchsia | Onagraceae | P | N | bird | bird | UV+Red | 0.138 | 49 | KL and SG dataset |
| *Fuchsia loxensis* Kunth | Fuchsia | Onagraceae | SP | N | bird | bird | UV+Pink | 0.086 | 2 | KL and SG dataset |
| *Fuchsia magdalenae* Munz | Fuchsia | Onagraceae | P | N | bird | bird | UV+Red | 0.131 | 2 | KL and SG dataset |
| *Fuchsia microphylla* Kunth | Fuchsia | Onagraceae | P | G | bird | bird | UV+Pink | 0.142 | 114 | KL and SG dataset |
| *Fuchsia paniculata* Lindl. | Fuchsia | Onagraceae | P | G | bird | bird | UV+Pink | 0.075 | 2 | KL and SG dataset |
| *Fuchsia regia* (Vand. ex Vell.) Munz | Fuchsia | Onagraceae | P | N | bird | bird | UV-Red | 0.081 | 253 | KL and SG dataset |
| *Fuchsia vulcanica* André | Fuchsia | Onagraceae | P | N | bird | bird | UV-Red | 0.07 | 2 | KL and SG dataset |
| *Oenothera lindheimeri* Engelm. & A.Gray | Oenothera | Onagraceae | P | EN | bee | bee | UV+Red | 0.042 | 126 | KL and SG dataset |
| *Calanthe vestita* Wall. ex Lindl. | Calanthe | Orchidaceae | P | EN | bee | bee | UV-White | 0.007 | 16 | KL and SG dataset |
| *Dendrobium nobile* Lindl. | Dendrobium | Orchidaceae | P | EN | bee | bee | UV-Pink | 0.052 | 16 | KL and SG dataset |
| *Oncidium ornithorhynchum* Kunth | Oncidium | Orchidaceae | P | N | bee | bee | UV-Pink | 0.052 | 216 | KL and SG dataset |
| *Eschscholzia californica* Cham. | Eschscholzia | Papaveraceae | P | EN | bee | bee | UV-Yellow | 0.032 | 280 | KL and SG dataset |
| *Lamprocapnos spectabilis* (L.) Fukuhara | Lamprocapnos | Papaveraceae | P | EN | bee | bee | UV+Pink | 0.11 | 257 | KL and SG dataset |
| *Papaver rhoeas* L. | Papaver | Papaveraceae | P | EN | bee | bee | UV-Red | 0.055 | 1 | Coimbra et al. 2020 |
| *Passiflora edulis* Sims | Passiflora | Passifloraceae | P | N | bee | bee | UV-White | 0.038 | 255 | KL and SG dataset |
| *Sesamum indicum* L. | Sesamum | Pedaliaceae | P | G | bee | bee | UV+White | 0.05 | 164 | KL and SG dataset |
| *Lapageria rosea* Ruiz & Pav*.* | Lapageria | Philesiaceae | P | N | bird | bird | UV+Pink | 0.128 | 112 | KL and SG dataset |
| *Mimulus cardinalis* Douglas ex Benth. | Mimulus | Phrymaceae | P | EN | bird | bird | UV+Red | 0.097 | 245 | KL and SG dataset |
| *Digitalis purpurea* L. | Digitalis | Plantaginaceae | P | EN | bee | bee | UV+Pink | 0.051 | 210 | KL and SG dataset |
| *Nanorrhinum scoparium* (Brouss. ex Spreng.) Yousefi & Zarre | Nanorrhinum | Plantaginaceae | P | EN | bee | bee | UV-Yellow | 0.027 | 1 | Coimbra et al. 2020 |
| *Penstemon pinifolius* Greene | Penstemon | Plantaginaceae | P | EN | bird | bird | UV+Red | 0.099 | 184 | KL and SG dataset |
| *Russelia equisetiformis* Schltdl. & Cham. | Russelia | Plantaginaceae | P | EN | bird | bird | UV-Red | 0.035 | 182 | KL and SG dataset |
| *Cantua buxifolia* Juss. ex Lam. | Cantua | Polemoniaceae | P | G | bird | bird | UV+Pink | 0.12 | 61 | KL and SG dataset |
| *Polygala myrtifolia* L. | Polygala | Polygalaceae | P | EN | bee | bee | UV+Pink | 0.143 | 2 | KL and SG dataset |
| *Eichhornia azurea* (Sw.) Kunth | Eichhornia | Pontederiaceae | P | N | bee | bee | UV-Blue | 0.292 | 117 | KL and SG dataset |
| *Cyclamen persicum* Mill. | Cyclamen | Primulaceae | P | EN | bee | bee | UV-White | 0.008 | 1 | Coimbra et al. 2020 |
| *Banksia prionotes* Lindl. | Banksia | Proteaceae | ST | EN | bird | bird | UV+Red | 0.063 | 35 | KL and SG dataset |
| *Grevillea banksii* R.Br. | Grevillea | Proteaceae | P | G | bird | bird | UV-Red | 0.04 | 148 | KL and SG dataset |
| *Leucospermum glabrum* E. Phillips | Leucospermum | Proteaceae | ST | EN | bird | bird | UV+Yellow | 0.079 | 205 | KL and SG dataset |
| *Telopea speciosissima* (Sm.) R. Br. | Telopea | Proteaceae | B | EN | bird | bird | UV+Red | 0.123 | 232 | KL and SG dataset |
| *Aconitum napellus* L. | Aconitum | Ranunculaceae | P | EN | bee | bee | UV+Blue | 0.089 | 6 | KL and SG dataset |
| *Anemone nemorosa* L*.* | Anemone | Ranunculaceae | P | EN | bee | bee | UV-White | 0.009 | 1 | Coimbra et al. 2020 |
| *Chaenomeles japonica* (Thunb.) Lindl. ex Spach | Chaenomeles | Rosaceae | P | EN | bird | bird | UV-Red | 0.081 | 69 | KL and SG dataset |
| *Eriobotrya japonica* (Thunb.) Lindl. | Eriobotrya | Rosaceae | P | EN | bee | bee | UV-White | 0.035 | 213 | KL and SG dataset |
| *Potentilla heptaphylla* L. | Potentilla | Rosaceae | P | EN | bee | bee | UV+Yellow | 0.081 | 1 | Coimbra et al. 2020 |
| *Galium verum* L. | Galium | Rubiaceae | P | EN | bee | bee | UV-Yellow | 0.015 | 1 | Coimbra et al. 2020 |
| *Hoffmannia refulgens* (Hook.) Hemsl. | Hoffmannia | Rubiaceae | P | N | bee | bee | UV+Red | 0.117 | 17 | KL and SG dataset |
| *Manettia cordifolia* Mart. | Manettia | Rubiaceae | P | N | bird | bird | UV+Red | 0.245 | 132 | KL and SG dataset |
| *Pentas lanceolata* (Forssk.) Deflers | Pentas | Rubiaceae | P | EN | bee | bee | UV-Red | 0.06 | 255 | KL and SG dataset |
| *Psychotria nuda* (Cham. & Schltdl.) Wawra | Psychotria | Rubiaceae | P | N | bird | bird | UV-Yellow | 0.027 | 45 | KL and SG dataset |
| *Citrus aurantium* L*.* | Citrus | Rutaceae | P | G | bee | bee | UV-White | 0.019 | 75 | KL and SG dataset |
| *Erythrochiton brasiliensis* Nees & Mart. | Erythrochiton | Rutaceae | P | N | bird | bird | UV+White | 0.109 | 192 | KL and SG dataset |
| *Paullinia cupana* Kunth | Paullinia | Sapindaceae | P | G | bee | bee | UV-White | 0.032 | 248 | KL and SG dataset |
| *Freylinia lanceolata* (L.) G.Don | Freylinia | Scrophulariaceae | P | EN | bee | bee | UV-Yellow | 0.044 | 214 | KL and SG dataset |
| *Scrophularia xanthoglossa* Boiss. | Scrophularia | Scrophulariaceae | P | EN | bee | bee | UV-Black | 0.037 | 1 | Coimbra et al. 2020 |
| *Lycium shawii* Roem. & Schult. | Lycium | Solanaceae | P | EN | bee | bee | UV-White | 0.018 | 1 | Coimbra et al. 2020 |
| *Nicotiana rustica* L*.* | Nicotiana | Solanaceae | P | G | bee | bee | UV+Yellow | 0.1 | 17 | KL and SG dataset |
| *Schizanthus pinnatus* Ruiz & Pav. | Schizanthus | Solanaceae | P | N | bee | bee | UV-White | 0.029 | 4 | KL and SG dataset |
| *Solanum betaceum* Cav. | Solanum | Solanaceae | P | N | bee | bee | UV-White | 0.06 | 102 | KL and SG dataset |
| *Streptosolen jamesonii* (Benth.) Miers | Streptosolen | Solanaceae | P | N | bird | bird | UV-Red | 0 | 116 | KL and SG dataset |
| *Strelitzia reginae* Banks | Strelitzia | Strelitziaceae | B | EN | bird | bird | UV+Red | 0.068 | 168 | KL and SG dataset |
| *Camellia japonica* L*.* | Camellia | Theaceae | P | EN | bird | bird | UV+Pink | 0.138 | 55,56 | KL and SG dataset |
| *Camellia sasanqua* Thunb. | Camellia | Theaceae | P | EN | bird | bird | UV-Pink | 0.033 | 56 | KL and SG dataset |
| *Camellia sinensis* (L.) Kuntze | Camellia | Theaceae | P | G | bee | bee | UV+White | 0.047 | 17 | KL and SG dataset |
| *Vellozia candida* J.C.Mikan | Vellozia | Velloziaceae | P | N | bee | bee | UV-White | 0.008 | 270 | KL and SG dataset |
| *Duranta erecta* L. | Duranta | Verbenaceae | P | EN | bee | bee | UV-Blue | 0.248 | 237 | KL and SG dataset |
| *Verbena bonariensis* L. | Verbena | Verbenaceae | P | EN | bee | bee | UV+Pink | 0.081 | 255 | KL and SG dataset |
| *Qualea multiflora* Mart. | Qualea | Vochysiaceae | P | N | bee | bee | UV+White | 0.259 | 212 | KL and SG dataset |
| *Alpinia purpurata* (Vieill.) K.Schum. | Alpinia | Zingiberaceae | B | G | bird | bird | UV-Red | 0.06 | 24 | KL and SG dataset |
| *Alpinia zerumbet* (Pers.) B.L.Burtt & R.M.Sm. | Alpinia | Zingiberaceae | P | EN | bee | bee | UV+Yellow | 0.06 | 8 | KL and SG dataset |
| *Curcuma longa* L. | Curcuma | Zingiberaceae | P | G | bee | bee | UV+White | 0.134 | 100 | KL and SG dataset |

**References**

1- Arnold SE, Faruq S, Savolainen V, McOwa PW, Chittka L. 2010. FReD: the floral reflectance database—a web portal for analyses of flower colour. *PloS one*, 5: e14287.

2- Burr B, Barthlott, W. 1993. Untersuchungen zur Ultraviolettreflexion von Angiospermenblüten: Magnoliidae, Ranunculidae, Hamamelididae, Caryophyllidae, Rosidae. *Tropische und subtropische Pflanzenwelt*.87: 193pp.

3- Coimbra G, Araujo C, Bergamo PJ, Freitas L, Rodríguez-Gironés MA. 2020. Flower conspicuousness to bees across pollination systems: a generalized test of the bee-avoidance hypothesis. *Frontiers in Plant Science*, 11: 558684.

4- Pérez F, Arroyo MT, Medel R, Hershkovitz MA. 2006. Ancestral reconstruction of flower morphology and pollination systems in *Schizanthus* (Solanaceae). *American Journal of Botany*, 93:1029-1038.

5- Bosch M, Simon J, Molero J, Blanché C. 2001. Breeding systems in tribe Delphinieae (Ranunculaceae) in the western Mediterranean area. *Flora*, 196: 101-113.

6- Sowig P. 1989. Effects of flowering plant's patch size on species composition of pollinator communities, foraging strategies, and resource partitioning in bumblebees (Hymenoptera: Apidae). *Oecologia*, 78: 550-558.

7- Stratton R. 1939. The Flowering Behavior of the Hollyhock (*Althea Rosea* Cav.). *In Proceedings of the Oklahoma Academy of Science*. 61-64.

8- Krieck CC, inatto T, Muller TS, Guerra M.P, Orh AI. 2008. Biologia reproductiva de *Alpinia zermbet*(Pers.) B.L. Burtt & R.M. Sm. (Zingiberaceae) em Florianopoli, Santa Catarina. *Revista Brasileira de Plantes Medicinals*, 10: 103-110.

9- Silva Ana PTP, de Faria PG. 2019. Biologia reprodutiva de *Aechmea blanchetiana* (Baker) Lb Sm.(Bromeliaceae) em um fragmento urbano de floresta atlântica do município de Juiz de Fora, Minas Gerais. *Diversidade e Gestão*, 3: 63-70.

10- Melo GFA, Machado IC, Lucen O. 1999. Reproducion de tres especies de *Clidemia* (Melastomataceae) en Brasil. *Revista de Biologia Tropical* 47: 359–363.

12- Steiner J, Zillikens A, Kamke R, Feja EP, de Barcellos Falkenberg D.2010. Bees and melittophilous plants of secondary Atlantic forest habitats at Santa Catarina Island, southern Brazil. *Oecologia Australis*, 14: 16-39.

13- Siqueira Filho JA, Tabarelli M.2006. Bromeliad species of the Atlantic forest of north-east Brazil: losses of critical populations of endemic species. Oryx, 40: 218-224.

15- Canela, M. B. F, Sazima, M.2003. *Aechmea pectinata*: a hummingbird dependent bromeliad with inconspicuous flowers from the rainforest in south eastern Brazil. *Annals of Botany*, 92: 731-737

16- Biedinger N, Barthlott W, Mainz Akademie der Wissenschaften und der Literatur.1993. Untersuchungen zur Ultraviolettreflexion von Angiospermenblüten I: Monocotyledoneae. Akademie der Wissenschaften und der Literatur.

17- Burr B, Rosen D, Barthlott W.1995. Untersuchungen zur Ultraviolettreflexion von Angiospermenbluten. III. Dilleniidae und Asteridae. *Tropische und subtropische Pflanzenwelt*, 93: 186pp.

19- de Araújo LDA, Quirino ZGM, Machado IC.2011. Reproductive phenology, floral biology and pollination of *Allamanda blanchetii*, an Apocynaceae endemic of the Caatinga. *Revista Brasileira de Botânica* 34: 211-222.

21- Hurrell JA, Keller HA, Krauczuk ER .2013. *Allamanda schottii* (Apocynaceae): nueva cita para la Flora Argentina. *Bonplandi*a, 5-10.

22- Botes C, Johnson SD, Cowling RM. 2008. Coexistence of succulent tree aloes: partitioning of bird pollinators by floral traits and flowering phenology. *Oikos* 117: 875-882.

23- Botes C. 2007. Reproductive co-existence among five sympatric single-stemmed aloes in the Gamtoos River Valley, Eastern Cape. Unpublished M. Sc. Thesis. Nelson Mandela Metropolitan University, Port Elizabeth.

24- Broglio-Micheletti SMF, Diniz MCC, Dias NDS, de Araújo AMN, Girón-Pérez K, Madalena JADS .2011. Insectos asociados a *Alpinia purpurata* (vieill.) k. schum.(Zingiberaceae) en Maceió y Rio Largo (AL), Brasil. *Revista Caatinga*, 24: 1-8.

27- Cestari C.2009. Epiphyte plants use by birds in Brazil. *Oecologia Brasiliensis*, 689-712.

29- Ulbricht MS .2006. Blütenökologie und Reproduktionsbiologie von fünf Waldrand- und Sekundärwaldarten des Atlantischen Regenwaldes, Pernambuco, Brasilien. Diploma Thesis, Ulm University, Ulm.

33- *Ivey CT, Martinez P, Wyatt R.2003.* Variation in pollinator effectiveness in swamp milkweed, *Asclepias incarnata* (Apocynaceae). *American Journal of Botany*, 90:214-225.

34- Díaz Lifante Z.1996. Reproductive biology of *Asphodelus aestivus* (Asphodelaceae). *Plant Systematics and Evolution* 200: 177-191.

35- Collins BG, Rebelo T.1987. Pollination biology of the Proteaceae in Australia and southern Africa. *Australian Journal of Ecology,* 12: 387-421.

40- Pemberton RW, Wheeler GS.2006. Orchid bees don't need orchids: evidence from the naturalization of an orchid bee in Florida. *Ecology*, 87: 1995-2001.

41- Vázquez DP, Simberloff D. 2004. Indirect effects of an introduced ungulate on pollination and plant reproduction. *Ecology Monographs*, 74:281–308.

42- Specht CD.2006. Systematics and evolution of the tropical monocot family Costaceae (Zingiberales): a multiple dataset approach. *Systematic Botany*, 31: 89-106.

43- Browning A, Smitley D, Studyvin J, Runkle ES, Huang ZY, Hotchkiss E. 2023. Variation in pollinator visitation among garden cultivars of marigold, portulaca, and bidens. *Journal of Economic Entomology*, 116: 872-881.

44- Wisdom MM, Richardson MD, Karcher DE, Steinkraus DC, McDonald GV.2019. Flowering persistence and pollinator attraction of early-spring bulbs in warm-season lawns. *HortScience*, 54: 1853-1859.

45- Sazima I, Buzato S, Sazima M.1995. The saw-billed hermit *Ramphodon naevius* and its flowers in southeastern Brazil. *Journal für ornithologie*, 136: 195-206.

47- Klinkhamer PG, de Jong TJ. 1990. Effects of plant size, plant density and sex differential nectar reward on pollinator visitation in the protandrous *Echium vulgare* (Boraginaceae). Oikos, 399-405.

49- Lomáscolo SB, MonmanyAC, Magro J, Suárez P, Andrada F. 2019. Effect of water increment on phenology, productivity, and herbivory of *Fuchsia boliviana* C.(Onagraceae) in Northwestern Argentina. *Ecología Austral*, 29: 185-193.

51- Gottsberger G.1986. Some pollination strategies in neotropical savannas and forests. *Plant Systematics and Evolution*, 152: 29-45.

52- Janeček Š, Bartoš M, Njabo KY 2015. Convergent evolution of sunbird pollination systems of Impatiens species in tropical Africa and hummingbird systems of the New World. *Biological Journal of the Linnean Society*, 115: 127-133.

55- Kunitake YK, Hasegawa M, Miyashita T, Higuchi H.2004. Role of a seasonally specialist bird *Zosterops japonica* on pollen transfer and reproductive success of *Camellia japonica* in a temperate area. *Plant Species Biology*, 19: 197-201.

56- Yumoto T.1987. Pollination systems in a warm temperate evergreen broad‐leaved forest on Yaku Island. *Ecological Research*, 2:133-145.

57- Hoffmann F.2005. Biodiversity and pollination: Flowering plants and flower-visiting insects in agricultural and semi-natural landscapes.

58- Hingston A. B.2005. Does the introduced bumblebee, *Bombus terrestris* (Apidae), prefer flowers of introduced or native plants in Australia? *Australian Journal of Zoology*, 53: 29-34.

59- Ren MX, Tang JY.2010. Anther fusion enhances pollen removal in *Campsis grandiflora*, a hermaphroditic flower with didynamous ss. *International Journal of Plant Sciences*, 171:275-282.

60- Ollerton J, Cranmer L, Stelzer R, Sullivan S, Chittka L.2008. Bird pollination of Canary Island endemic plants. *Nature Precedings*, 1-1.

61- Harborne JB, Smith DM.1978. Correlations between anthocyanin chemistry and pollination ecology in the Polemoniaceae. *Biochemical Systematics and Ecology*, 6:127-130.

62- Hanley ME, Awbi AJ, Franco M. 2014. Going native? Flower use by bumblebees in English urban gardens. *Annals of Botany*, 113: 799-806.

66- Colwell RK, Betts BJ, Bunnell P, Carpenter FL, Feinsinger P. 1974. Competition for the nectar of *Centropogon valerii* by the hummingbird *Colibri thalassinus* and the flower-piercer *Diglossa plumbea*, and its evolutionary implications. *The condor*, 76: 447-452.

69- Fang Q, Chen YZ, Huang SQ. 2012. Generalist passerine pollination of a winter-flowering fruit tree in central China. *Annals of botany*, 109(2), 379-384.

70- Goldblatt P, Manning JC.2006. Radiation of pollination systems in the Iridaceae of sub-Saharan Africa. *Annals of botany*, 97: 317-344

73- Petanidou T, Ellis WN. 1993. Pollinating fauna of a phryganicecosystem: composition and diversity. *Biodiversity Letters* 1: 9–22.

75- Karmakar P. 2013. Pollination biology of *Citrus aurantiifolia* (Christm.) Swingle: a medicinally important fruit plant*. International Journal of Innovative Research and Development*, 2: 138-142.

77- Pemberton RW, Liu H.2008. Naturalized orchid bee pollinates resin‐reward flowers in florida: novel and known mutualisms. *Biotropica*, 40:714-718.

78- Rivera GL.2000. Nuptial nectary structure of Bignoniaceae from Argentina. *Darwiniana,* 227-239.

80- Snow AA, Roubik DW.1987. Pollen deposition and removal by bees visiting two tree species in Panama. *Biotropica*, 57-63.

85- Córdoba SA, Cocucci AA.2011. Flower power: its association with bee power and floral functional morphology in papilionate legumes. *Annals of botany*, 108:919-931.

86- Murray KG, Feinsinger P, Busby WH, Linhart YB, Beach JH, Kinsman S.1987. Evaluation of character displacement among plants in two tropical pollination guilds. *Ecology*, 68: 1283-1293.

89- Marazzi B, Mangili S, Gygax A, Jousson A.2002. Biology and spread of the new alien species Coreopsis grandiflora (Asteraceae) in southern Switzerland. *Bollettino della Società ticinese di scienze naturali* .110:57-70

92- Kay KM, Reeves PA, Olmstead RG, Schemske DW.2005. Rapid speciation and the evolution of hummingbird pollination in neotropical *Costus* subgenus *Costus* (Costaceae): evidence from nrDNA ITS and ETS sequences. *American Journal of Botany*, 92: 1899-1910.

94- Kay KM, Schemske DW.2003. Pollinator assemblages and visitation rates for 11 species of Neotropical *Costus* (Costaceae). *Biotropica*, 35: 198-207;

95- Bricker JS.1992. Pollination biology of the genus *Crinodendron* (Elaeocarpaceae). *Journal of the Arizona-Nevada Academy of Science*, 51-54.

97- du Puy DJ, Labat JN, Rabevohitra R, Villiers JF, Bosser J, Moat J.2002. The leguminosae of Madagascar. The Leguminosae of Madagascar. *Curtis's Botanical Magazine*, 14: 231-240.

100- Kato M, Kosaka Y, Kawakita A, Okuyama Y, Kobayashi C, Phimminith T, Thongphan D.2008. Plant–pollinator interactions in tropical monsoon forests in Southeast Asia. *American Journal of Botany*, 95: 1375-1394.

102- Ramírez F, Kallarackal J. 2019. Tree tomato (*Solanum betaceum* Cav.) reproductive physiology: A review. *Scientia horticulturae*, 248: 206-215.

104- Armbruster WS, Webster GL. 1979. Pollination of two species of *Dalechampia* (Euphorbiaceae) in Mexico by euglossine bees. *Biotropica*, 278-283.

107- Martínez-Harms J, Palacios AG, Márquez N, Estay P, Arroyo MT, Mpodozis J. 2010. Can red flowers be conspicuous to bees? *Bombus dahlbomii* and South American temperate forest flowers as a case in point. *Journal of Experimental Biology*, 213: 564-571.

112- Humaña AM, Riveros M. 1994. Biologıa de la reproduccion en la especie trepadora *Lapageria rosea* R. et P. (Philesiaceae). *Gayana Botanica* 51: 49–55.

114- Arizmendi MC, Dominguez CA, Dirzo R.1996. The role of an avian nectar robber and of hummingbird pollinators in the reproduction of two plant species. *Functional Ecology*, 119-127.

116- Baker HG, Baker I, Hodges SA.1998. Sugar composition of nectars and fruits consumed by birds and bats in the tropics and subtropics 1. *Biotropica*, 30: 559-586.

117- Barrett SC.1978. Floral biology of *Eichhornia azurea* (Swartz) Kunth (Pontederiaceae). *Aquatic Botany*, 5: 217-228.

121- Bittencourt Jr NS, Semir J.2004. Pollination biology and breeding system of *Zeyheria montana* (Bignoniaceae). *Plant Systematics and Evolution*, 247: 241-254.

122- Boechat RF, da Silva BF, Nunes-Freitas AF.2019. Bird-epiphyte interactions in three Atlantic Forest environments in southeastern Brazil. *Revista Brasileira de Ornitologia*, 27: 108-114.

126- Carr BL, Gregory DP, Raven PH, Tai W.1986. Experimental hybridization and chromosomal diversity within *Gaura* sect. Gaura (Onagraceae). *Systematic botany*, 98-111.

129- Collins BG, Newland C, Briffa P.1984. Nectar utilization and pollination by Australian honeyeaters and insects visiting *Calothamnus quadrifidus* (Myrtaceae). *Australian Journal of Ecology*, 9: 353-365.

132- Consolaro H, Silva EBD, Oliveira PED.2005. Variação floral e biologia reprodutiva de *Manettia cordifolia* Mart. (Rubiaceae). *Brazilian Journal of Botany*, 28: 85-94.

137- Cutting EM.1921. On the Pollination Mechanism of Incarvillea, Delavayi, Franch. *Annals of Botany*, 1: 63-71.

140- Dupont YL, Skov C.2004. Influence of geographical distribution and floral traits on species richness of bees (Hymenoptera: Apoidea) visiting *Echium* species (Boraginaceae) of the Canary Islands. *International Journal of Plant Sciences*, 165: 377-386.

143- Etcheverry AV, Alemán CET.2005. Reproductive Biology of *Erythrina falcata* (Fabaceae: Papilionoideae) 1. *Biotropica*, 37: 54-63.

146- Fonseca LC, Vizentin-Bugoni J, Rech AR, Alves, MAS.2015. Plant-hummingbird interactions and temporal nectar availability in arestinga from Brazil. *Anais da Academia Brasileira de Ciências*, 87: 2163-2175.

148- Herscovitch JC, Martin AR. 1989. Pollen-pistil interactions in *Grevillea banksii*: The Pollen Grain, Stigma, Transmitting Tissue and in Vitro Pollinations. *Grana*, 28: 69-84.

149- Forster PI.1994. Diurnal insects associated with the flowers of *Gomphocarpus physocarpus* E. Mey.(Asclepiadaceae), an introduced weed in Australia. *Biotropica*, 214-217.

150- Free JB.1966. The pollination requirements of broad beans and field beans (*Vicia faba*). *The Journal of Agricultural Science*, 66: 395-397.

154- García-Franco JG, Burgoa DM, Pérez TM.2001. Hummingbird flower mites and *Tillandsia* spp. (Bromeliaceae): polyphagy in a cloud forest of Veracruz, Mexico. *Biotropica*, 538-542.

158- Gowda V, Kress WJ.2013. A geographic mosaic of plant–pollinator interactions in the Eastern Caribbean Islands. *Biotropica*, 45: 224-235.

164- Shankar U, Mukhtar Y. (2022). Diversity and foraging behaviour of pollinators on *Sesamum indicum L*. *Indian Journal of Ecology*, 49: 1984-1988.

168- Hoffmann F, Daniel F, Fortier A, Hoffmann-Tsay SS.2011. Efficient avian pollination of *Strelitzia reginae* outside of South Africa. *South African Journal of Botany*, 77: 503-505.

169- Iltis HH.1963. *Napaea dioica* (Malvaceae): whence came the type? *American Midland Naturalist*, 90-109.

170- Van Jaarsveld EJ, Ward-Hilhorst E. 1994. *Gasterias* of South Africa: A new revision of a major succulent group. Fernwood Press.

172- Johnston MO.1991. Pollen limitation of female reproduction in *Lobelia cardinalis* and *L. siphilitica*. *Ecology*, 1500-1503.

181-Kratochwil A, Beil M, Schwabe A.2009. Complex structure of pollinator-plant interaction-webs: random, nested, with gradients or modules? *Apidologie*, 40: 634-650.

182- Mendes RS, Araújo-Hoffmann FP. 2024. Nectar plants visited by hummingbirds in an urban area of southern Brazil. *Urban Ecosyst* 28: 1–13

183- Ladley JJ, Kelly D, Robertson AW.1997. Explosive flowering, nectar production, breeding systems, and pollinators of New Zealand mistletoes (Loranthaceae). *New Zealand Journal of Botany*, 35: 345-360.

184- Lange RS, Scobell SA, Scott PE.2000. Hummingbird-syndrome traits, breeding system, and pollinator effectiveness in two syntopic *Penstemon* species. *International Journal of Plant Sciences*, *161:* 253-263.

186- Lee B. 1966. Jamaican species of Emilia, cytogenetics and taxonomy. *Science News and Notes (The Journal of the Association of Science Teachers of Jamaica).* 2:14-15

189- Liu A.Z, Kress WJ, Wang H, Li DZ.2002. Insect pollination of *Musella* (Musaceae), a monotypic genus endemic to Yunnan, China. *Plant systematics and evolution*, 235:135-146.

190- Liu R, Gao Y, Fan Z, Wang X, Xiao J, Zhang Q.2020. Within-day temporal isolation of two species of Iris (Iridaceae) sharing the same pollinator. *Biological Journal of the Linnean Society*, 130: 447-457.

191- Locatelli E, Machado ICS.1999. Comparative study of the floral biology in two ornithophilous species of Cactaceae: *Melocactus zehntneri* and *Opuntia palmadora. Bradleya,* 1999: 75-85.

192- Lopes AV.2002. Polinização por beija-flores em remanescente da Mata Atlântica Pernambucana, Nordeste do Brasil. PhD Thesis, Universidade Estadual de Campinas, Campinas, Brazil.

195- Luo Z, Zhang D, Renner SS.2008. Why two kinds of ss in buzz‐pollinated flowers? Experimental support for Darwin's division‐of‐labour hypothesis. *Functional Ecology*, 22: 794-800.

197- Ribbens E, Anderson BA, Fant J. 2011. *Opuntia fragilis* (Nuttall) Haworth in Illinois: pad dynamics and sexual reproduction. *Haseltonia*, 2011: 67-78.

200- Marazzi B, Conti E, Endress PK.2007. Diversity in anthers and stigmas in the buzz-pollinated genus *Senna* (Leguminosae, Cassiinae). *International Journal of Plant Sciences*, 168: 371-391.

201- Martén‐Rodríguez S, Quesada M, Castro AA, Lopezaraiza‐Mikel M, Fenster CB.2015. A comparison of reproductive strategies between island and mainland Caribbean Gesneriaceae. *Journal of Ecology*, 103: 1190-1204.

202- Martinelli G.1995. Reproductive biology of Bromeliaceae in the Atlantic rainforest of southeastern Brazil. PhD Thesis, University of St Andrews.

203- Wester P, Claßen-Bockhoff R. 2006. Hummingbird pollination in *Salvia haenkei* (Lamiaceae) lacking the typical lever mechanism. *Plant Systematics and Evolution* 257: 133–146.

204- Rivera-Hutinel A, Bustamante RO, Marín VH, Medel R. 2012. Effects of sampling completeness on the structure of plant–pollinator networks. *Ecology*, 93: 1593-1603.

205- Johnson CM. 2015. Flowers with style: The role of pollinators in the origin and maintenance of Proteaceae diversity with a focus on the genus *Leucospermum.* Doctoral dissertation, Stellenbosch: Stellenbosch University.

206- Medan D, Montaldo NH.2005. Ornithophily in the Rhamnaceae: the pollination of the Chilean endemic *Colletia ulicina*. Flora-Morphology, Distribution, *Functional Ecology of Plants*, 200: 339-344.

207- Mendonça LB, Anjos LD.2005. Beija-flores (Aves, Trochilidae) e seus recursos florais em uma área urbana do Sul do Brasil. *Revista Brasileira de Zoologia*, 22: 51-59

209- Morton ES.1979. Effective pollination of *Erythrina fusca* by the Orchard Oriole (*Icterus spurius*): Coevolved behavioral manipulation? *Annals of the Missouri Botanical Garden*, 482-489.

210- Nazir R, Reshi Z, Wafai BA.2008. Reproductive ecology of medicinally important Kashmir Himalayan species of *Digitalis* L. *Plant species biology*, 23: 59-70.

212- Oliveira PE.1998. Reproductive biology, evolution and taxonomy of the Vochysiaceae in Central Brazil. Reproductive Biology (SJ Owens PJ Rudall, eds.). Kew, Royal Botanic Gardens, London, 381-393.

213- Ahmad S, Khalofah A, Khan SA, Khan KA, Jilani MJ, Hussain T, Ahmad Z. 2021. Effects of native pollinator communities on the physiological and chemical parameters of loquat tree (*Eriobotrya japonica*) under open field condition. *Saudi Journal of Biological Sciences*, 28: 3235-3241.

214- Manning JC, Maluleke R, Ebrahim I, Helme NA. 2021. The genus *Freylinia Pangella* ex Colla (Scrophulariaceae: Teedieae): a re-assessment of the systematics and conservation status. *South African Journal of Botany*, 142: 352-369.

216- Singer RB, Cocucci AA. 1999. Pollination mechanisms in four sympatric southern Brazilian Epidendroideae orchids*. Lindleyana* 14: 47–56.

215- Pammel LH.1888. On the pollination of *Phlomis tuberosa*, L, and the perforation of flowers. *Transactions of the St. Louis academy of science*, 5: 241-277

217- Parachnowitsch AL, Elle E.2005. Insect visitation to wildflowers in the endangered Garry Oak, Quercus garryana, ecosystem of British Columbia. *The Canadian Field-Naturalist*, 119:245-253.

218- Pawlikowski T, Biliñski M, Kosior A, Fijał J.2007. Site constancy of bumblebees (Hymenoptera: Apiformes: Bombus Latr.) in the habitats of two forest successional series of the Western Carpathians. *Journal of Apicultural Science*, 51: 109-117.

222- Pemberton RW, Liu H.2008. Potential of invasive and native solitary specialist bee pollinators to help restore the rare cowhorn orchid (*Cyrtopodium punctatum*) in Florida. *Biological Conservation*, 141:1758-1764.

223- Hung TT, Hsu HC, Kuo YF. 2019. Quantifying color and textural patterns of petals and studying their association with pollinators: using genus Sinningia (Gesneriaceae) as an example. In 2019 ASABE annual international meeting . American Society of Agricultural and Biological Engineers.

226- Pickens AL.1929. Bird pollination problems in California. *The Condor*, 31: 229-232..

228- Pinheiro M, Brito VLG, Sazima M.2018. Pollination biology of melittophilous legume tree species in the Atlantic Forest in Southeast Brazil. *Acta Botanica Brasilica*, 32: 410-425.

229- Tokuda N, Hattori M, Abe K, Shinohara Y, Nagano Y, Itino T. 2015. Demonstration of pollinator‐mediated competition between two native Impatiens species, *Impatiens noli-tangere and I. textori* (Balsaminaceae). *Ecology and Evolution*, 5: 1271-1277.

230- Raina R H, Saini M S, Khan ZH. 2019. Altitudinal food preference of bumblebee species (Hymenoptera: Apidae) from Indian Himalaya. *Journal of Entomology and Zoology Studies*, 7: 234-237.

231- Raguso RA, Pichersky E.1999. New Perspectives in Pollination Biology: Floral Fragrances. A day in the life of a linalool molecule: Chemical communication in a plant‐pollinator system. Part 1: Linalool biosynthesis in flowering plants. *Plant Species Biology*, 14:95-120.

232- Pyke GH.1981. Effects of inflorescence height and number of flowers per inflorescence on fruit set in waratahs (*Telopea speciosissima*). *Australian Journal of Botany*, 29:419-424.

234- Raina R, Gupta LM.1997. Increasing seed yield in glory lily (*Gloriosa superba*)-experimental approaches. In II WOCMAP Congress Medicinal and Aromatic Plants, Part 3: Agricultural Production, Post-Harvest Techniques, Biotechnology 502 (pp. 175-180).

236- Geerts S, Pauw A. 2011. Easy technique for assessing pollination rates in the genus Erica reveals road impact on bird pollination in the Cape fynbos, South Africa. *Austral Ecology*, 36: 656-662.

237- Reddy TB, Reddi CS.1996. Pollination ecology of *Duranta repens*(Verbenaceae). *Journal of the Bombay Natural History Society*, 93: 193-201.

238- Renner SS.1989. A survey of reproductive biology in Neotropical Melastomataceae and Memecylaceae. *Annals of the Missouri Botanical Garden*, 496-518.

242- Samanta A, Bera B, Karmakar P. 2023. Pollination ecology of an important medicinal plant *Hellenia speciosa* (J. Koenig) SR Dutta of Asiatic tropics. Biodiversitas *Journal of Biological Diversity*, 24.

244- Sazima M, Machado ICS.1983. Biologia floral de *Mutisia coccinia* St. Hil.(Asteraceae). *Revista Brasileira de Botânica*, 6: 103-108.

245- Schemske DW, Bradshaw Jr HD.1999. Pollinator preference and the evolution of floral traits in monkeyflowers (Mimulus). *Proceedings of the National Academy of Sciences*, 96: 11910-11915.

246- Schleuning M, Templin M, Huamán V, Vadillo GP, Becker T, Durka W, Markus F, Matthies, D.2011. Effects of inbreeding, outbreeding, and supplemental pollen on the reproduction of a hummingbird‐pollinated clonal Amazonian herb. *Biotropica*, 43:183-191.

248- Krug C, Garcia MVB, Gomes FB. 2015. A scientific note on new insights in the pollination of guarana (*Paullinia cupana var. sorbilis*). *Apidologie*, 46: 164-166.

249- Sergeant CA.2009. The influence of *S. frutescens* on adrenal cytochrome P450 11B-hydroxylase. PhD Thesis, Stellenbosch: University of Stellenbosch.

253- Snow DW, Teixeira DL.1982. Hummingbirds and their flowers in the coastal mountains of southeastern Brazil. *Journal für Ornithologie*, 123: 446-450.

255- Steiner J, Zillikens A, Kamke R, Feja EP, de Barcellos Falkenberg D.2010. Bees and melittophilous plants of secondary Atlantic forest habitats at Santa Catarina Island, southern Brazil. *Oecologia Australis*, 14: 16-39.

256- Mach BM, Potter DA. 2017. Woody ornamentals for bee‐friendly landscapes (Ohio Valley region). *Entomology Reports* 1.

257- Hodges, L. (2012). Bleeding heart: A review for growers. *HortTechnology*, 22: 517-522.

258- Buzato S, Sazima M, Sazima I. 2000. Hummingbird‐pollinated floras at three Atlantic Forest sites 1. *Biotropica*, 32: 824-841.259- Struck M.1997. Floral divergence and convergence in the genus *Pelargonium* (Geraniaceae) in southern Africa: ecological and evolutionary considerations. *Plant Systematics and Evolution*, 208: 71-97.

260- Suzuki K.1984. Pollination system and its significance on isolation and hybridization in Japanese Epimedium (Berberidaceae). *The botanical magazine= Shokubutsu-gaku-zasshi*, 97: 381-396.

261- Osada N, Sugiura S. 2006. Effects of pollinators and flower bud herbivores on reproductive success of two ericaceous woody species differing in flowering season. *Botan*y, 84: 112-119.

263- Tie S, He YD, Lázaro A, Inouye DW, Guo YH, Yang CF.2023. Floral trait variation across individual plants within a population enhances defense capability to nectar robbing. *Plant Diversity*, 45: 315-325.

264- Tobe H, Hakki MI, Langhammer L.1989. Floral nectary in *Medinilla magnifica*, an old world Melastomataceae. *Botanische Jahrbücher für Systematik,*111:57–62

265- Gentry AH. 1974. Coevolutionary patterns in Central American Bignoniaceae. *Ann. Missouri Bot. Gard.,* 61:728-759.

270- Verçoza FC.2012. Polinização de *Vellozia candida* Mikan (Velloziaceae) nos Afloramentos Rochosos do Costão de Itacoatiara, Niterói, RJ: Um Caso de Melitofilia em Inselbergue do Brasil*. EntomoBrasilis*, 5: 29-32.

271- Costa RP. 2019. The pollinator community of the Madeiran endemic *Echium candicans*: individual-based network metrics, relation with plant traits, and pollinator behavior.Master's thesis, Universidade de Lisboa (Portugal).

272- Vieira RE, Kotaka CS, Mitsui MH, Taniguchi AP, de Toledo VAA, Ruvolo-Takasusuki MCC, Terada Y, Sofia SH, Costa FM.2002. Biologia floral e polinização por abelhas em siratro (Macroptilium atropurpureum Urb.). *Acta Scientiarum*. 24: 857-861.

274- Vogel S, Westerkamp C, Thiel B, Gessner, K.1984. Ornithophily on the Canary Islands. *Plant Systematics and Evolution*, 146: 225-248.

275- Wester P, Claßen-Bockhoff R.2007. Floral diversity and pollen transfer mechanisms in bird-pollinated Salvia species. Annals of Botany, 100:401-421.

276- Wester P, Claßen-Bockhoff R. 2011. Pollination Syndromes of New World Salvia Species with Special Reference to Bird Pollination1. *Annals of the Missouri Botanical Garden*, 98:101-155.

277- Wester P, Cairampoma L, Haag S, Schramme J, Neumeyer C, Claßen-Bockhoff R.2020. Bee exclusion in bird-pollinated *Salvia* flowers: the role of flower color versus flower construction. *International Journal of Plant Sciences*, 181: 770-786.

278- Williams NH, Dressler RL.1976. Euglossine pollination of Spathiphyllum (Araceae). *Selbyana*, 1:349-356.

280- Wojcik VA, Frankie GW, Thorp RW, Hernandez JL.2008. Seasonality in bees and their floral resource plants at a constructed urban bee habitat in Berkeley, California. *Journal of the Kansas Entomological Society*, 81: 15-28.

Table S2. Evolutionary models with AIC and transition rates values. To perform this reconstruction, we first analyse which of the following evolutionary models best fits the data on “white-red” flowers: ER (equal rates), ARD (all different rates), and SYM (symmetrical). The best model was selected by comparing Akaike weights (AICc) and ΔAIC. The values of transition rates are: W (white), R (red) and O (Other) and the trace between them indicates the direction of change of state. Highlighted in gray shade is the selected model.

|  | **AICc** | **ΔAIC** | **Red -White** | **White-Red** | **Red-Other** | **Other- Red** | **White-Other** | **Other-White** |
| --- | --- | --- | --- | --- | --- | --- | --- | --- |
| **ER** | 711.7734 | 9.7248 | 1.000239 | 1.000239 | 1.000239 | 1.000239 | 1.000239 | 1.000239 |
| **ARD** | 702.0486 | 0.0000 | 0.921324 | 1.212737 | 0.190734 | 0.139850 | 0.00000 | 0.00000 |
| **SYM** | 702.8541 | 0.8054 | 0.013642 | 0.013642 | 0.057296 | 0.057296 | 0.037301 | 0.037301 |

Table S3. AICc values of evolutionary models with values of transition rates. To perform this reconstruction, we first analyse which of the following evolutionary models best fits the data on “yellow” flowers: ER (equal rates), ARD (all different rates), and SYM (symmetrical). The best model was selected by comparing Akaike weights (AICc) and ΔAIC. The values of transition rates are: Y (yellow) and O (Other) and the trace between them indicates the direction of change of state. Highlighted in gray shade is the selected model.

|  | **AICc** | **ΔAIC** | **Yellow -Other** | **Other-Yellow** |
| --- | --- | --- | --- | --- |
| **ER** | 332.0395 | 38.753 | 0.00508 | 0.00508 |
| **ARD** | 293.2862 | 0.0000 | 0.186498 | 0.037749 |
| **SYM** | 332.0395 | 38.753 | 0.00508 | 0.00508 |

Table S4. Results of the analysis of variance (ANOVA type II) for generalized linear models (GLM) adjusted to evaluate the effect of pollinator type (bee vs. bird) and hue category (red, white, yellow, and others) on flower chromatic contrast. Two models were tested: one considering the response to chromatic contrast in the bee visual system (Model I) and another considering the response to chromatic contrast in the bird visual system (Model II). Significance codes: *** p < 0.001; ** p < 0.01; * p < 0.05; p < 0.10.

| **Model** | **Variables** | **χ²** | **Df** | **Pr(>Chisq)** |
| --- | --- | --- | --- | --- |
| **I Chromatic Contrast in the visual system of bees** | Pollinator type | 4.3980 | 1 | 0.0359805 * |
|  | Hue category | 19.5212 | 3 | 0.0002133 *** |
|  | Pollinator type vs. Hue category | 0.6385 | 3 | 0.8875758 |
| **II Chromatic Contrast in the visual system of birds** | Pollinator type | 2.469 | 1 | 0.11612 |
|  | Hue category | 41.796 | 3 | 4.433^e-09^ *** |
|  | Pollinator type vs. Hue category | 7.671 | 3 | 0.05332 |

Table S5. Comparisons of estimated marginal means for combinations of flower hue categories (red, white, yellow, other) in the generalized linear model (GLM) for chromatic contrast in the bee visual system. Highlighted in bold is the comparison where the *p*-values are statistically significant.

| **Hue category** | **Estimate** | **SE** | ***p*** |
| --- | --- | --- | --- |
| Other - Red | 0.393 | 0.145 | **0.0357** |
| Other - White | 0.206 | 0.148 | 0.5057 |
| Other - Yellow | -0.234 | 0.171 | 0.5174 |
| Red - White | -0.187 | 0.146 | 0.5773 |
| Red - Yellow | -0.628 | 0.169 | **0.0015** |
| White - Yellow | -0.441 | 0.172 | **0.0538** |

Table S6. Comparisons of estimated marginal means for combinations of pollinator type (bees vs. birds) and flower hue categories (red, white, yellow, other) in the generalized linear model (GLM) for chromatic contrast in the bird visual system. Highlighted in bold is the comparison where the p-values are statistically significant.

| **Pollination system** | **Hue category** | **Estimate** | **SE** | **t** | ***p*** |
| --- | --- | --- | --- | --- | --- |
| Bee x Bee | Other - Red | -0.1178 | 0.1245 | -0.946 | 0.9809 |
|  | Other - White | 0.1575 | 0.0845 | 1.864 | 0.5773 |
|  | Other - Yellow | -0.0659 | 0.1009 | -0.653 | 0.9980 |
|  | Red - White | 0.2754 | 0.1227 | 2.245 | 0.3304 |
|  | Red - Yellow | 0.0520 | 0.1345 | 0.386 | 0.9999 |
|  | White - Yellow | -0.2234 | 0.0986 | -2.265 | 0.3189 |
| Bee x Bird | Other - Other | 0.1450 | 0.1245 | 1.164 | 0.9409 |
|  | Other - Red | -0.2479 | 0.0816 | -3.038 | **0.0542** |
|  | Other - White | 0.5057 | 0.1287 | 3.929 | **0.0030** |
|  | Other - Yellow | -0.0141 | 0.1545 | -0.091 | 1.0000 |
|  | Red - Red | -0.1301 | 0.1207 | -1.078 | 0.9607 |
|  | Red - White | 0.6235 | 0.1564 | 3.985 | **0.0024** |
|  | Red - Yellow | 0.1037 | 0.1782 | 0.582 | 0.9991 |
|  | White - White | 0.3481 | 0.1269 | 2.743 | 0.1167 |
|  | White - Yellow | -0.1716 | 0.1530 | -1.122 | 0.9514 |
|  | Yellow - Yellow | 0.0518 | 0.1626 | 0.318 | 1.0000 |
| Bird x Bee | Other - Red | -0.2628 | 0.1530 | -1.718 | 0.6759 |
|  | Other - White | 0.0126 | 0.1227 | 0.102 | 1.0000 |
|  | Other - Yellow | -0.2108 | 0.1345 | -1.567 | 0.7690 |
|  | Red - White | 0.4055 | 0.0788 | 5.145 | **<.0001** |
|  | Red - Yellow | 0.1821 | 0.0962 | 1.893 | 0.5575 |
|  | White - Yellow | -0.5715 | 0.1384 | -4.130 | **0.0014** |
| Bird x Bird | Other - Red | -0.3929 | 0.1207 | -3.255 | **0.0288** |
|  | Other - White | 0.3607 | 0.1564 | 2.306 | 0.2962 |
|  | Other - Yellow | -0.1590 | 0.1782 | -0.892 | 0.9865 |
|  | Red - White | 0.7536 | 0.1250 | 6.027 | **<.0001** |
|  | Red - Yellow | 0.2339 | 0.1514 | 1.544 | 0.7823 |
|  | White - Yellow | -0.5198 | 0.1812 | -2.868 | 0.0852 |

Table S7. Comparisons of estimated marginal means for combinations of pollinator system and hue categories (white-red flowers subset) in the generalized linear mixed model (GLMM) fitted to investigate the interactive effects of pollinator type and hue category on UV reflection optima phenotype values (θ).

| **Pollination system** | **Hue category** | **Estimate** | **SE** | **t** | ***p*** |
| --- | --- | --- | --- | --- | --- |
| Bee x Bee | Other - Red | -0.03401 | 0.000994 | -34.208 | <.0001 |
|  | Other - White | 0.01185 | 0.000994 | 11.921 | <.0001 |
|  | Red - White | 0.04586 | 0.000994 | 46.129 | <.0001 |
| Bird x Bird | Other - White | 0.02515 | 0.000994 | 25.297 | <.0001 |
|  | Other - Red | 0.00899 | 0.000994 | 9.045 | <.0001 |
|  | Red - White | 0.01616 | 0.000994 | 16.252 | <.0001 |
| Bee x Bird | Other - Red | 0.0045 | 0.000994 | 4.53 | <.0001 |
|  | Other - Other | -0.00449 | 0.000994 | -4.514 | <.0001 |
|  | Other - White | 0.02066 | 0.000994 | 20.782 | <.0001 |
|  | Red - Red | 0.03851 | 0.000994 | 38.738 | <.0001 |
|  | White - White | 0.00881 | 0.000994 | 8.861 | <.0001 |
|  | Red - White | 0.05467 | 0.000994 | 54.99 | <.0001 |
| Bird x Bee | Other - Red | -0.02952 | 0.000994 | -29.693 | <.0001 |
|  | Other - White | 0.01634 | 0.000994 | 16.436 | <.0001 |
|  | Red - White | 0.00735 | 0.000994 | 7.391 | <.0001 |

Table S8. Comparisons of estimated marginal means for combinations of pollinator system and hue categories (yellow flowers subset) in the generalized linear mixed model (GLMM) fitted to investigate the interactive effects of pollinator type and hue category on UV reflection optima phenotype values (θ). Highlighted in bold is the comparison where the p-values are statistically significant.

| **Pollination system** | **Hue category** | **Estimate** | **SE** | ***p*** |
| --- | --- | --- | --- | --- |
| Bee x Bee | Other - Yellow | -0.00703 | 0.00232 | **0.0133** |
| Bird x Bird | Other - Yellow | -0.00038 | 0.00232 | 0.9984 |
| Bee x Bird | Other - Other | -0.00251 | 0.00232 | 0.7018 |
|  | Other - Yellow | -0.00289 | 0.00232 | 0.599 |
|  | Yellow - Yellow | 0.00414 | 0.00232 | 0.2823 |
| Bird x Bee | Other - Yellow | -0.00452 | 0.00232 | 0.2094 |
