# Supplementary material for: Evolution of UV reflection in bee‐ and bird‐pollinated flowers
Source: Plant Biol (Stuttg). 2025 Nov 14;28(1):201–14. doi: 10.1111/plb.70138 (PMC12710832; doi:10.1111/plb.70138)
Supplement: Supplementary file 2 — Fig. S1. Smoothed average reflectance spectra of flower colours from the species analysed. The colours of the flowers were categorized based on their reflection in the UV (300‐400 nm), blue (400‐500 nm), green (500‐600 nm) and red (600–700) wavelengths. Established thresholds for mean spectral reflection were 0.1 (10%) for UV, 0.3 (30%) for blue, 0.4 (40%) for green, 0.5 (50%) to distinguish green flowers from blue and red ones, and 0.6 (60%) for red (adapted from Camargo et al. 2019 and Coimbra et al. 2020). The red line highlights the UV threshold (0.1) to differentiate reflectance patterns of UV versus non‐UV flowers. Reflectance patterns average of non‐UV (UV−) colours are represented on the left, and those of UV (UV+) colours are represented on the right. Fig. S2. Reflectance spectra of red, yellow, and white flowers pollinated by bees (left) and birds (right) from the analysed species. The red line indicates the UV threshold (0.1), used to distinguish UV‐reflective (UV+) from non‐UV‐reflective (UV−) flowers. Fig. S3. Estimated Marginal Means of chromatic contrast based on the bee visual system, considering (A) pollinator type (bee vs. bird) and (B) flower hue categories (red, white, yellow, and others). The points represent the mean estimated marginal means for each pollinator type (A) and hue category (B), while the error bars indicate the 95% confidence intervals. The figure highlights potential differences in chromatic contrast across hue categories and as perceived by different types of pollinators Full statistical results are provided in Tables S7 and S8. Fig. S4. Estimated Marginal Means of chromatic contrast based on the bird visual system as a function of pollinator type (bee vs. bird) and flower hue category (white, red, yellow, other). The points represent the mean estimated marginal means for each combination of pollinator type and hue category. The graph highlights how chromatic contrast varies across different hue categories depending on the polli [file PLB-28-201-s002.docx]

**Supplementary figures**

**
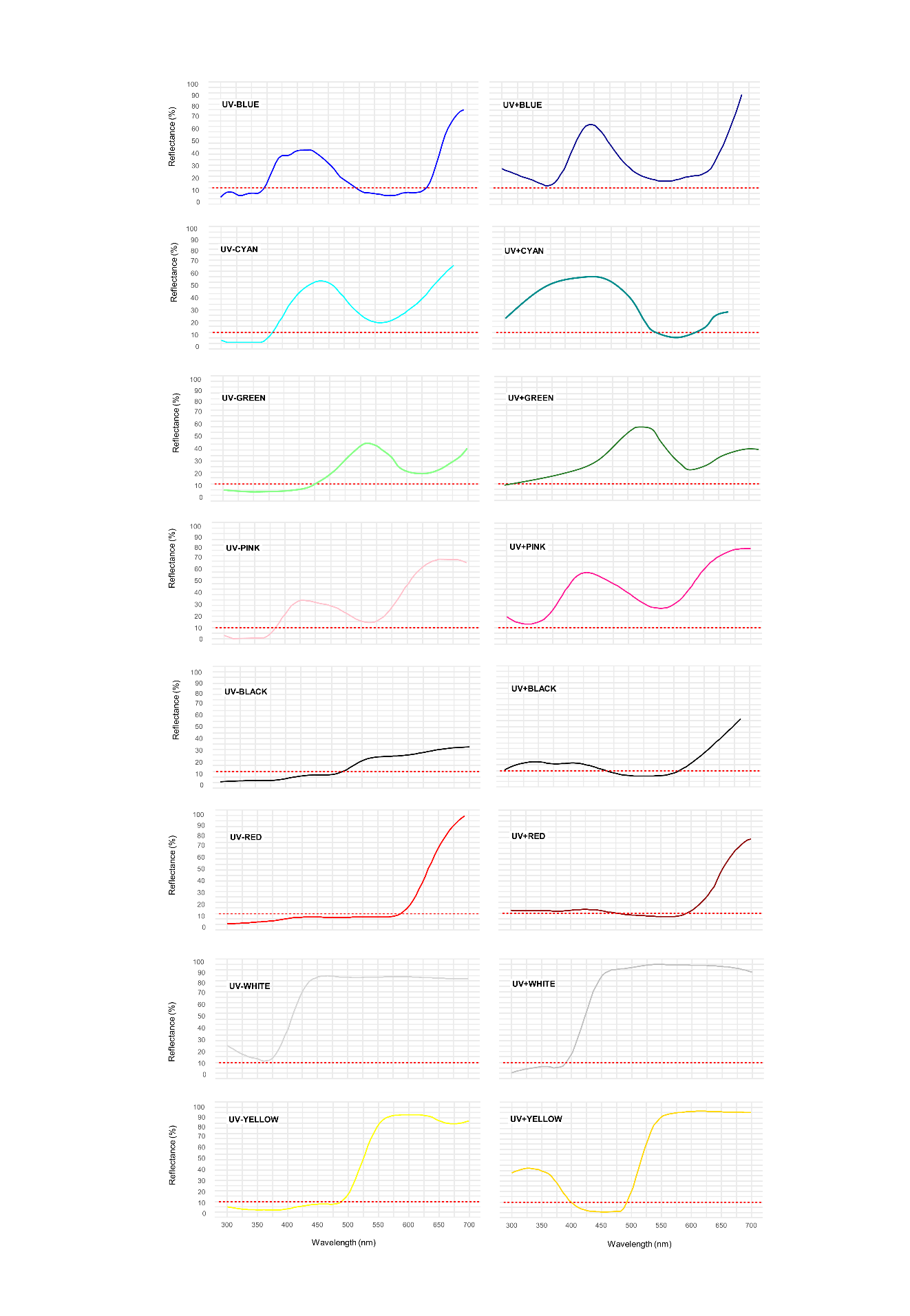
**

Figure S1. Smoothed average reflectance spectra of flower colors from the species analysed. The colors of the flowers were categorized based on their reflection in the UV (300-400nm), blue (400-500nm), green (500-600nm) and red (600-700) wavelengths. Established thresholds for mean spectral reflection were 0.1 (10%) for UV, 0.3 (30%) for blue, 0.4 (40%) for green, 0.5 (50%) to distinguish green flowers from blue and red ones, and 0.6 (60%) for red (adapted from Camargo et al., 2019 and Coimbra et al., 2020). The red line highlights the UV threshold (0.1) to differentiate reflectance patterns of UV vs. non-UV flowers. Reflectance patterns average of non-UV (UV-) colors are represented on the left, and those of UV (UV+) colors are represented on the right.

**
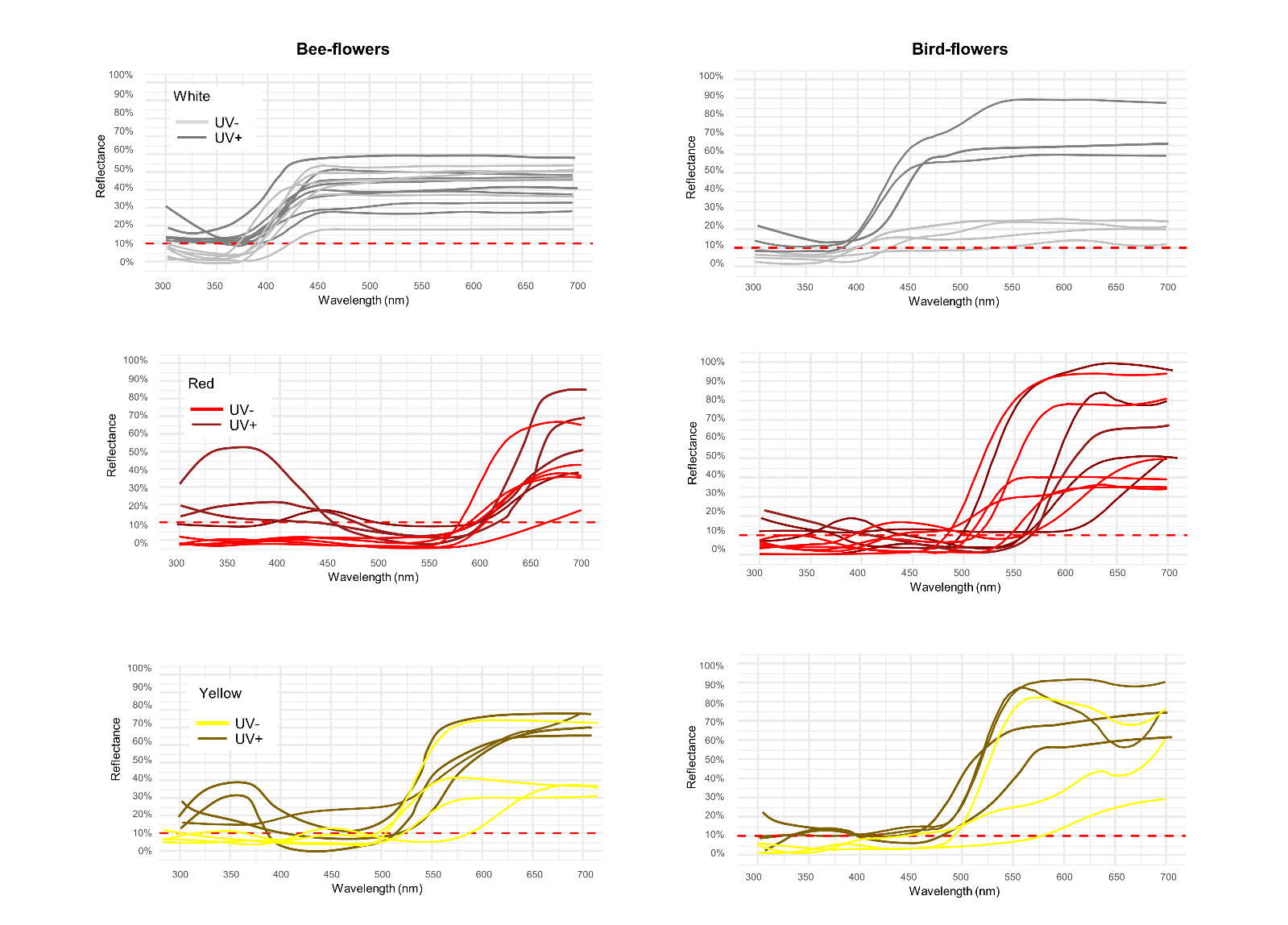
**

Figure S2. Reflectance spectra of red, yellow, and white flowers pollinated by bees (left) and birds (right) from the analyzed species. The red line indicates the UV threshold (0.1), used to distinguish UV-reflective (UV+) from non-UV-reflective (UV-) flowers.


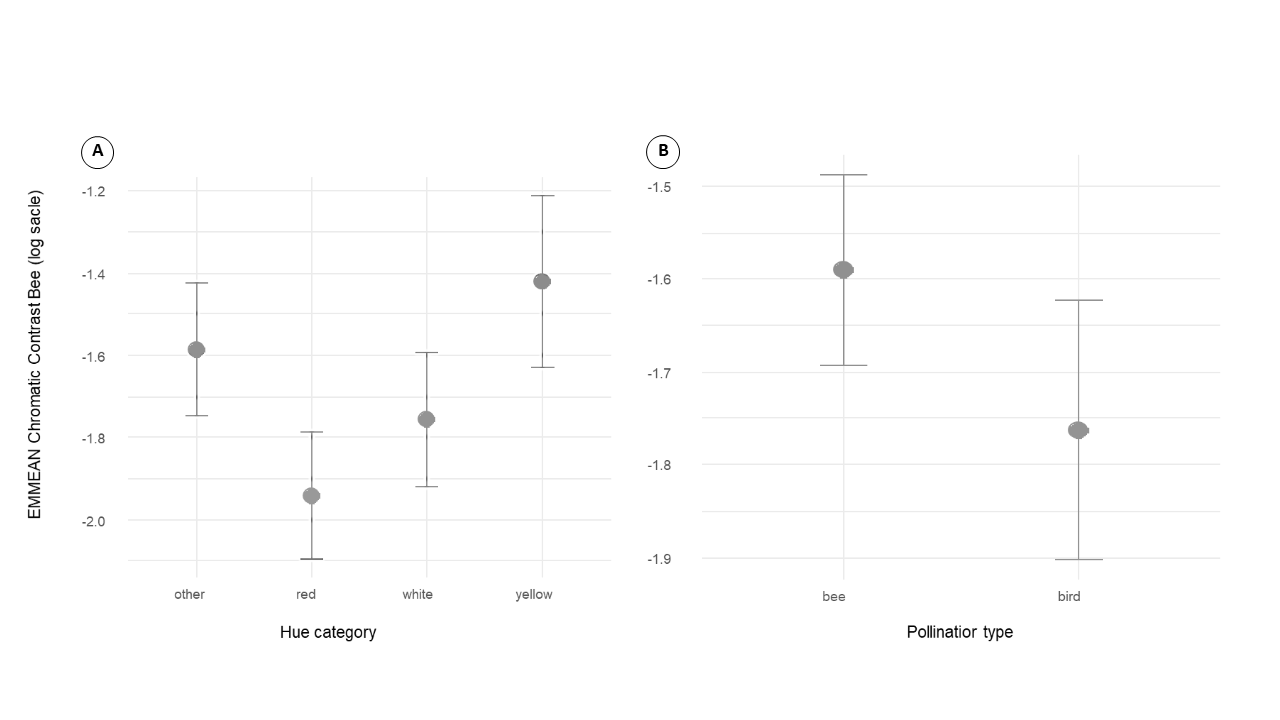


Figure S3. Estimated Marginal Means of chromatic contrast based on the bee visual system, considering (A) pollinator type (bee *vs.* bird) and (B) flower hue categories (red, white, yellow, and others). The points represent the mean estimated marginal means for each pollinator type (A) and hue category (B), while the error bars indicate the 95% confidence intervals. The figure highlights potential differences in chromatic contrast across hue categories and as perceived by different types of pollinators Full statistical results are provided in Supplementary Table S7–S8.

**
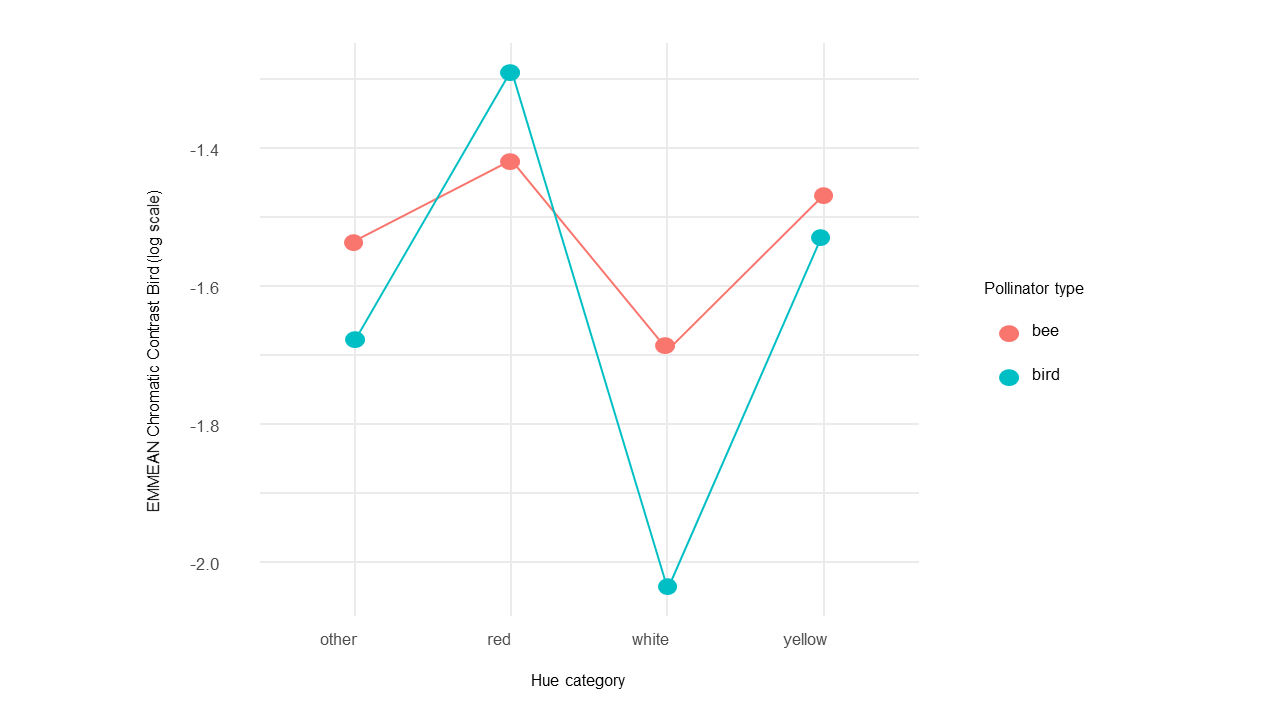
**

Figure S4. Estimated Marginal Means of chromatic contrast based on the bird visual system as a function of pollinator type (bee *vs.* bird) and flower hue category (white, red, yellow, other). The points represent the mean estimated marginal means for each combination of pollinator type and hue category. The graph highlights how chromatic contrast varies across different hue categories depending on the pollinator type. Full statistical results are provided in Supplementary Table S7–S8.


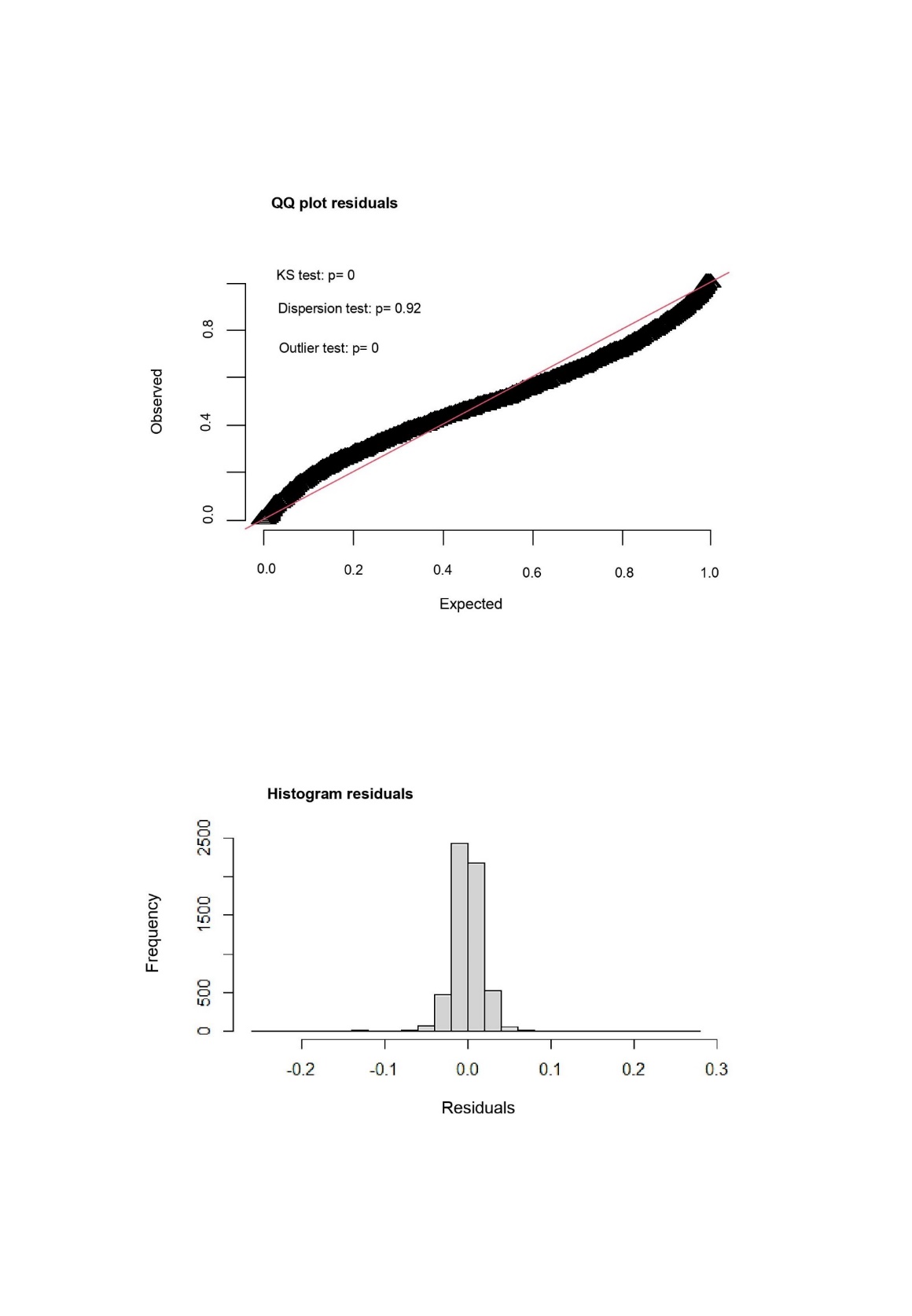


Figure S5. Residual Diagnostics for the GLMM investigating the interactive effects of pollinator type (bee vs bird) and hue category on optima phenotype (θ) of white-red flowers. Additionally, the results of Kolmogorov-Smirnov (KS), dispersion and outlier statistical tests are provided.


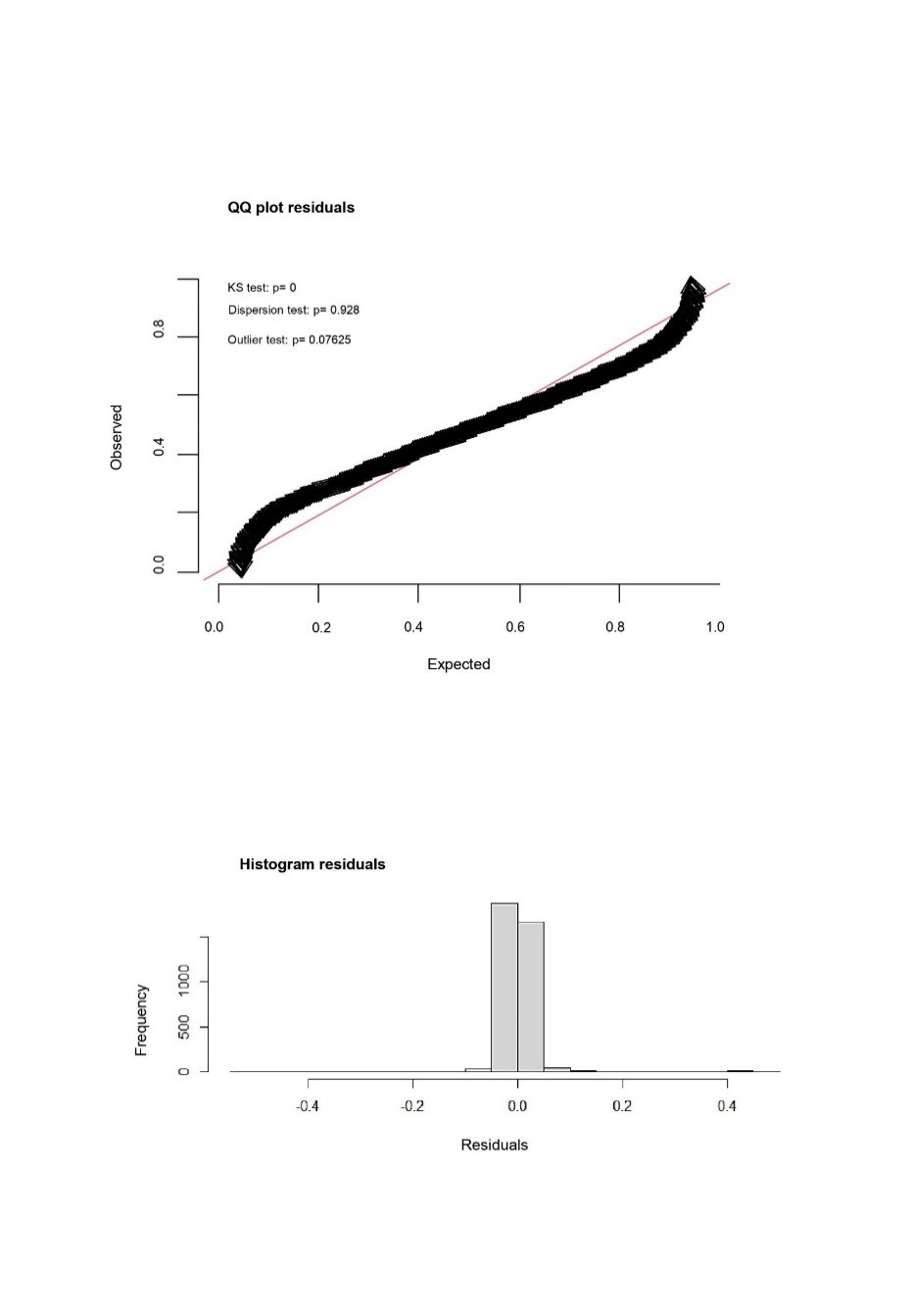


Figure S6. Residual Diagnostics for the GLMM investigating the interactive effects of pollinator type (bee vs bird) and hue category on optima phenotype (θ) of yellow flowers. Additionally, the results of Kolmogorov-Smirnov (KS), dispersion and outlier statistical tests are provided.
